# Impressive near-infrared brightness and singlet oxygen generation from strategic lanthanide–porphyrin double-decker complexes in aqueous solution

**DOI:** 10.1038/s41377-019-0155-9

**Published:** 2019-05-22

**Authors:** Jing-Xiang Zhang, Wai-Lun Chan, Chen Xie, Yan Zhou, Ho-Fai Chau, Partha Maity, George T. Harrison, Aram Amassian, Omar F. Mohammed, Peter A. Tanner, Wai-Kwok Wong, Ka-Leung Wong

**Affiliations:** 1 Department of Chemistry, Hong Kong Baptist University, Kowloon Tong, Hong Kong S.A.R., China; 2 Hanshan Normal University, Chaozhou, Guangdong Province China; 3 Department of Biology, Hong Kong Baptist University, Kowloon Tong, Hong Kong S.A.R., China; 4 KAUST Solar Center, Division of Physical Science and Engineering, King Abdullah University of Science and Technology (KAUST), Thuwal, 23955-6900 Saudi Arabia

**Keywords:** Optical materials and structures, Optical spectroscopy

## Abstract

Although lanthanide double-decker complexes with hetero-macrocyclic ligands as functional luminescent and magnetic materials have promising properties, their inferior water solubility has negated their biomedical applications. Herein, four water-soluble homoleptic lanthanide (**Ln** **=** **Gd**, **Er**, **Yb** and **La**) sandwiches with diethylene-glycol-disubstituted porphyrins (**DD**) are reported, with their structures proven by both quantum chemical calculations and scanning tunneling microscopy. Our findings demonstrate that the near-infrared emission intensity and singlet oxygen (^1^O_2_) quantum yields of **YbDD** and **GdDD** in aqueous media are higher than those of the reported capped lanthanide monoporphyrinato analogues, **YbN** and **GdN**; the brightness and luminescence lifetime in water of **YbDD** are greater than those of **YbN**. This work provides a new dimension for the future design and development of molecular theranostics-based water-soluble double-decker lanthanide bisporphyrinates.

## Near-infrared devices: Water-soluble probes use rare earths to brighten bioimaging

Tweaking the chemical structure of naturally occurring rings called porphyrins has improved the biocompatibility of molecular probes that emit bright light at low doses. Recent studies have shown that rare earths can act as potent sources of near-infrared light when they are attached to porphyrin ‘antennas’ that transfer absorbed light energy to the metal. Ka-Leung Wong from Hong Kong Baptist University and co-workers have now developed porphyrin—rare earth complexes that can easily enter aqueous environments such as bodily fluids. The team synthesized water-friendly porphyrins by adding short diethylene glycol chains to the ring framework, and used them to trap rare earths including ytterbium into sandwich-shaped arrangements. Characterizations revealed the new complexes retained their efficient, long-lasting light emissions in aqueous solutions, and could act as generators of electronically excited oxygen for photodynamic therapy.

## Introduction

**Fig. 1:**
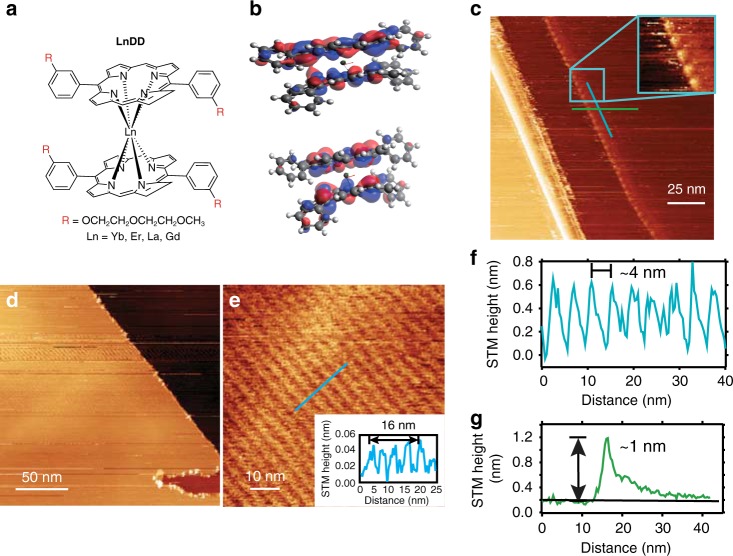
Structure characterization. **a** Molecular structures of water-soluble lanthanide porphyrin complexes: use of porphyrins with double-decker forms with DEG-chain-conjugated porphyrins. **b** Calculated LUMO (top) and HOMO (bottom) by applying density functional theory (sidechains not included); STM images for **YbDD** drop-casting onto HOPG: **c** toluene 1 mmol 5 μL, 140 × 140 nm^2^, −1.5 V sample bias, 50 pA, (inset) blue box: a high-magnification image, cyan and green lines denote the height line profiles (Fig. 1f, g); **d** chloroform 1 mmol 25 μL 270 × 270 nm^2^ image, +2 V sample bias, 100 pA, **e** 75 × 75 nm^2^, image +2 V sample bias, 100 pA, (inset) blue height profile across rows. **f** Line profile from Fig. 1c along the row of features. **g** Line profile across the HOPG step edge, the height of the feature ~1 nm (bottom terrace) is indicated

Near-infrared (NIR) luminescent lanthanide materials have been widely utilized and increasingly researched in telecommunications engineering, laser technology, and biomedical science by virtue of their extraordinary photophysical properties^1–5^. However, challenges remain that lanthanides are intrinsically constrained by the Laporte-forbidden 4*f*–4*f* transitions that render their direct excitation rather inefficient^6,7^. To circumvent this issue, *π*-conjugated hetero-macrocycles, such as porphyrins, possessing (i) high-absorption cross-sections, (ii) triplet states resonating well with lanthanide absorption bands, and (iii) four “hard” nitrogen donor atoms matching “hard” lanthanides, have become promising antenna in use for optimal energy sensitization and protective coordination^8–14^. Sandwich-type lanthanide–porphyrin complexes can afford more preferable or even surprising emission results, given that double-decker lanthanide complexes have recently spanned the fields of electrochromic/optoelectronic devices, photovoltaic cells, single-molecule magnets, and even molecular rotors—though with few bio-related counterparts^15–18^. Despite their well-characterized, long-lived NIR emission, and ^1^O_2_ generation, most lanthanide–macrocycle complexes suffer from inferior water solubility that considerably hampers their further development in biomedical fields^17,18^. Tarakanova et al. performed the first comprehensive study on double-decker lanthanide complexes and investigated their interaction with water. Unfortunately, only one incorporated water molecule was considered and only intramolecular hydrogen bonding was examined, without the description of an aqueous solution^19^. Recently, water-soluble gadolinium–porphyrin complexes were reported by Zang et al.^20^. Their complexes have two porphyrin rings but do not form a sandwich structure. Therefore, their recorded molar extinction coefficients and singlet oxygen quantum yield in water were much lower than those of our Gd-analogues, although these two series of complexes both have similar two porphyrin rings as antenna chromophores. The porphyrin structure is rigid and its excited energy can be nonradiatively transferred to an acceptor^21^. Thus, we recently focused on and have already reported three water-soluble (up to 1 μM), polyethylene glycol (PEG) chain-conjugated, capped lanthanide monoporphyrinates of (i) organelle specificity, **YbRhB**^22^, (ii) tumor selectivity, **YbN**^23^, and (iii) photodynamic therapy, **GdN**^24^. Herein, we introduce four water-soluble porphyrin-based lanthanide double-decker complexes (**LnDD**, where Ln = La, Er, Gd and Yb, Fig. 1a) with remarkable NIR photophysical properties in aqueous solution. Upon the strategic installation of two optimally short hydrophilic methylated diethylene glycol (DEG) chains on the tailor porphyrin **Por(2DEG)** for sandwich lanthanide complexation, **YbDD** exhibited improved NIR luminescence quantum yield and lifetime in water and outperformed previously reported **YbN**. The singlet oxygen generation efficiency in terms of quantum yield (Φ_O2_) of **GdDD** was measured to be slightly higher than that of **GdN**. Our findings substantiate the hypotheses that the double-decker complexation between porphyrins can (i) facilitate better lanthanide sensitization in the presence of two antenna chromophores rather than one and (ii) minimize the innersphere quenching effect by lowering the number of bound water molecules under the macrocyclic sandwich design. This work provides unique results for the photophysical data of **LnDD** in aqueous media and more importantly, a new dimension for the future design and development of molecular theranostics-based water-soluble double-decker lanthanide bisporphyrinates. Structural elucidation in this study was performed using various techniques, as described herein, since it is difficult to prepare single crystals suitable for X-ray analysis.

## Results

### Structural characterization by calculation

The porphyrin dianion unit is planar with perpendicular aromatic rings (Supplementary Fig. S31). The structures of lanthanide double-decker porphyrins have been previously discussed in the literature^25,26^. The schematic structure of the double-decker complexes is depicted in Fig. 1a. The skeleton structure, without DEG sidechains and with or without a negative charge, was optimized by calculation (refer to Section 5, SI) because the total charge depends upon the pH and solvent. The structure optimization of **YbDD** and **[YbDD]**^**−**^ using MOPAC^27^ in the LUMPAC 1.3.0^28,29^ suite of programs is shown in Supplementary Fig. S32a, b, and is similar to that using the ORCA^30^ program (Supplementary Fig. S33) and Firefly QC^31^ package (Supplementary Figs. S35 and S37), which is partially based upon the GAMESS (US)^32^ source code. The porphyrin ring system is no longer planar due to (i) the cation-π attractive forces and (ii) π–π repulsive forces. The N–N distance from the bottom to the top of the double decker is comparable with the distances within each sandwich layer. The structure was also optimized for the AlDD system (Supplementary Fig. S36) and shows six short Al–N bonds and two longer bonds. These bonds give rise to a distorted structure. The calculated highest occupied molecular orbital/lowest unoccupied molecular orbital (HOMO/LUMO) are also given for **LnDD** in Fig. 1b and Supplementary Fig. S34 (DEG chains are omitted for clarity).

### Structural characterization by scanning tunneling microscopy

Scanning tunneling microscopy (STM) is an advanced technique that can be used for probing molecular assemblies on an individual molecule basis. The study of porphyrins assembly and structure at the vacuum and liquid interface on surfaces is relatively advanced^33^. In particular, several studies have been undertaken on double-decker structured molecules^34,35^. **YbDD** was deposited on a clean highly oriented pyrolytic graphite (HOPG) (0001) surface by placing a drop of dilute solution and evaporating at room temperature. The molecules formed self-assembled motifs without further treatment through surface adsorption and diffusion^33^. The STM topographic image in Fig. 1c shows a high-magnification image of a region of a drop-cast surface with additional features decorating the step edges. As shown in the zoom inset, these form a ~4 nm periodic row of separation, and an apparent height of ~1 nm (line profile Fig. 1g) is present. This height, which was recorded at −1.5 V filled state, is strongly influenced by the electronic effects of both the tip apex and molecular surface junctions^36,37^. In a different trial with **YbDD**, a close-packed arrangement was observed and is shown in Fig. 1d, e. This ordered arrangement is long-ranged ~100 nm and aligned parallel to the HOPG step edge direction. The features also show a separation of ~4 nm in the direction perpendicular to the step direction (parallel unresolved) as indicated by the height line profile in Fig. 1e inset. Therefore, a templating effect originating at the step is suggested. The overall behavior of the drop-cast double-decker **YbDD** on HOPG is in line with previous studies of double-decker motifs and porphyrin ligands, with a favorable interaction and ability to spontaneously form a periodic assembly. The large ~4 nm spacing between resolvable features is consistent with literature accounts of similarly structured molecules with the spacing correlated to alkyl chain length^34,38^ with individual molecules packing face-on with the oxy-alkyl chain R groups having a favorable arrangement on the HOPG surface, leading to the observed spacing. We attribute the observed features, rows and protrusions (Fig. 1c, e) to single molecules with further work underway to resolve the exact inner-molecular structure.

### Structural characterization by nuclear magnetic resonance

**Fig. 2:**
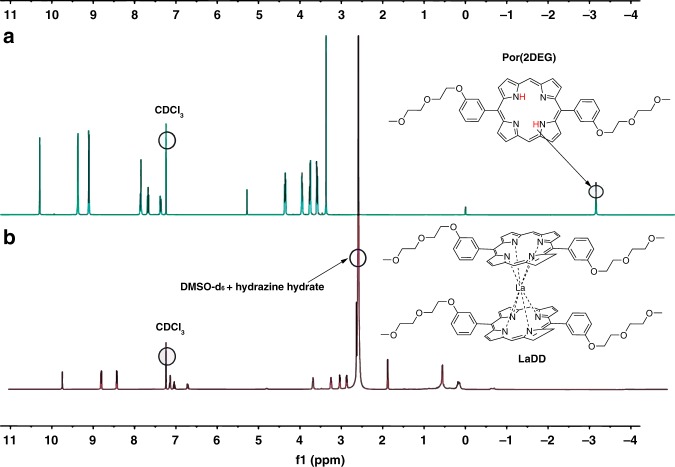
Formation of double-decker structure visualized by NMR spectra. Room-temperature NMR spectra of **a** the ligand **Por(2DEG)** in CDCl_3_ and **b** **LaDD** in 1:1 CDCl_3_: DMSO-*d*_6_ mixed with 1% hydrazine hydrate

The synthesis and characterization of the double-decker porphyrinate lanthanide complexes with Ln = La, Er, Gd, and Yb trivalent ions are shown in Supplementary Scheme S1, Supplementary Figs. S1–S10 and Supplementary Table S1. Due to the paramagnetic properties of the latter three lanthanide ion complexes, **LaDD** was synthesized as the analogue for nuclear magnetic resonance (NMR) analysis. Upon the addition of hydrazine hydrate, a well-resolved **LaDD** NMR spectrum could be obtained (Fig. 2) because hydrazine hydrate served as a reducing agent and assisted the formation of monoanionic diamagnetic complexes^9^. The protons of the single ligand **Por(2DEG)** can be categorized into peripheral and internal. The peripheral aromatic protons are typically located approximately at 6.5–10.0 ppm, while the DEG sidechain aliphatic protons normally lie within the range of 1.5–4.0 ppm. The peak of the hydrazine hydrate mixed with DMSO-*d*_6_ is observed at 2.6 ppm. The ring current effect strongly shifts the two internal protons on the porphyrin upfield to −3.2 ppm. The disappearance of internal N–H peaks and the proton shifting can then serve as an indication of metallization with the lanthanide ion. No signal is observed in the negative range (equated to internal N–H protons) in the spectrum of **LaDD**, while all peaks are subjected to upfield shifting due to the anisotropy of the *f*-metal ion as well as the impact of lanthanide-induced shifts^10^. It is noted that the theoretically most possible supramolecular trimers or even multiple aggregate structures can also give rise to similar NMR spectra, but the high-resolution mass spectra (HRMS) and STM images corroborate the double-decker structure of **LaDD** (and thus the **LnDD** series) unambiguously (Supplementary Fig. S6).

### Photophysical studies and brightness

**Fig. 3:**
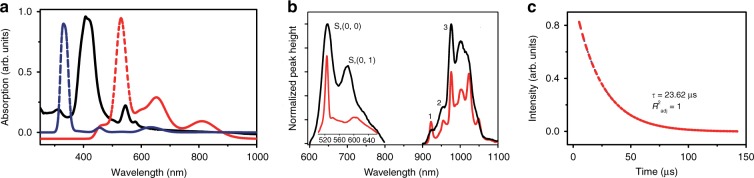
Photophysical properties of the Yb(III) complexes. **a** Room temperature absorption spectrum of **YbDD** (black solid line) and calculated spectra using half-height widths of 1500 cm^−1^ using LUMPAC (blue dashed line) and ORCA (red dashed line) programs (refer to Supplymental Information Section 4). **b** Comparison of the emission bands of **YbN** (black) and **YbDD** (red) at 298 K in aqueous solution (*λ*_ex_ = 425 nm). **c** Decay curve of Yb^3+^ emission in Fig. 3b (blue points) and fitted curve (red dashed line) using a monoexponential function

Photophysical properties of LnDD (Ln = Yb, Er, Gd, La) have been measured (Supplementary Figs. S20–S24) and summarized in Supplementary Table S3. Upon photoexcitation at 425 nm (representing the strongest absorption band, the B or Soret band, Fig. 3a, black experimental spectrum), **YbDD** showcases superior photophysical performance compared with the monoporphyrinato counterpart/analogue **YbN** (serving as the control) under various solvent systems (Supplementary Figs. S11–14 and Supplementary Table S2). The NIR quantum yields of **YbDD** were measured by comparison with the standard **YbTPP(Tp)**, which was reported as 3.2% in dichloromethane with the same excitation wavelength of 425 nm^39^. The NIR emission quantum yields of **YbDD** in toluene (water) were recorded as 3.5% (2.8%), while those of **YbN** in these solvents were 2.8% (2.7%), shown in Table 1. To explain these results, firstly, **YbDD**, which has two antenna ligand groups, should transcend **YbN**, which has only one. As shown in Fig. 3b, the emission spectrum of **YbDD** comprises several parts: the porphyrin ligand visible-NIR emission and the Yb^3+^ (^2^F_5/2_ → ^2^F_7/2_) NIR emission, which has an equal peak height in this figure. The peaks at 647 and 699 nm represent the porphyrin fluorescence from the Q-band singlet nominally labeled S_1_. The S_2_ singlet (B-band) emission is also observed at a much weaker intensity and at shorter wavelengths (not shown). From the comparison with the low temperature 77 K emission spectrum of **YbDD** (Supplementary Fig. S19), the hot emission bands 1, 2, and 3 in Fig. 3b may correspond to the transitions from the three excited states of ^2^F_5/2_, and the energy intervals between band 3 (975 nm; 10,260 cm^−1^) and bands 4–6 in Supplementary Fig. S19 can identify the three levels above the ground state energy of ^2^F_7/2_. The energy transfer from the porphyrin ligands to the Yb^3+^ ion is observed to be efficient because the metal ion is not excited by 425 nm radiation in the absence of an antenna. However, the presence of both ligand fluorescence and lanthanide emission at room temperature suggests that the energy transfer rate from the porphyrin to Yb^3+^ is similar to the nanosecond regime. The lower emission quantum yield of **YbDD** in water than that in toluene is attributable to the quenching by high-frequency O–H vibrations. The trivalent lanthanide ions belong to the hard Lewis acid category with the coordination number of up to 8–12 so that under saturation of the lanthanides’ inner coordination sphere by ligands offers vacancies for solvent molecule coordination^35^. The **YbN** system was confirmed to have unsaturated seven-coordinated Yb^3+^: four N from the porphyrin ring and three O from the Kläui [(η^5^-C_5_H_5_) Co{(MeO)_2_P = O}_3_]^−^ anion capped oxygen atoms. To shield the Yb^3+^ ion in an aqueous environment and suppress luminescence quenching, the double-decker complexation strategy in **YbDD** fulfills the eight-coordination number requirement.

**Table 1:** Luminescence lifetime (*τ*), quantum yield (*Φ*) and brightness (BR) of **YbDD** and **YbN** in toluene or water

	*τ* (µs)^a^	*τ* (μs)^a^	*Φ* (%)^b^	*Φ* (%)^b^	BR^c^
	Toluene	H_2_O	Toluene	H_2_O	H_2_O
**YbDD**	28.19	23.62	3.5	2.8	2540
**YbN**	23.02	12.04	2.8	2.7	1850

^a^Determined from the emission decay curve monitored at *λ*_em_ = 978 nm (^2^F_5/2_ → ^2^F_7/2_) with *λ*_ex_ = 425 nm (Conc.: 1 μM)

^b^The relative quantum yields of the Yb^3+^ emission (λ_ex_ = 425 nm) from the two Yb^3+^ complexes in toluene and H_2_O were obtained by comparison with the standard **YbTPP(Tp)**

^c^Calculated from BR = attenuation coefficient × quantum yield; molar attenuation coefficients were obtained from the absorption spectra in water at 425 nm by applying the Beer–Lambert Law

Brightness is the product of quantum yield and molar attenuation coefficient^40,41^, and demonstrates the radiant energy emitted per frequency interval unit area per solid angle. For bioimaging purposes where low dosage is preferred because of adverse effects, the brightness is a more superior indicator of applicability than quantum yield, since, with higher brightness, low-abundance fluorescent compounds are detected more easily. The brightness of **YbDD** exceeds that of **YbN** by a factor of 1.37 (Table 1). The NIR ^2^F_5/2_ → ^2^F_7/2_ emission lifetimes of **YbDD** and **YbN** were determined to be 23.6 μs in water (Fig. 3c and Supplementary Fig. S17, S18) and 28.2 μs in toluene, which are both higher than the values for **YbN** (Table 1). **YbDD** shows a longer ^2^F_5/2_ → ^2^F_7/2_ lifetime, which mainly results from its higher symmetry than **YbN**. With a more symmetric structure, the *f-f* transition mechanism is of less forced electric dipole character and more vibronic, and the lifetime for a specific transition is longer^42^. This trend is consistent with the measured NIR emission quantum yields in water and toluene. It is worth noting that most porphyrin-based NIR dyes for biological applications have little emission in the NIR-II biological window because no metal ion is coordinated. Furthermore, the maximal absorption peaks of these dyes are usually located only from 650 to 800 nm^43^. Both of these reasons limit the application prospects. One commercially available NIR-II dye (NIR-II dye #900883, Sigma-Aldrich) has a similar emission peak located at 1050 nm, which is the same as that of **YbDD**, but its NIR emission quantum yield is ~2%, which is lower than that of **YbDD**. The impressive NIR emission quantum yields and long NIR emission lifetime of **YbDD** in aqueous solution, together with its hydrophilic property, hold tremendous promise as a (NIR) bioimaging probe.

### Singlet-oxygen generation

**Fig. 4:**
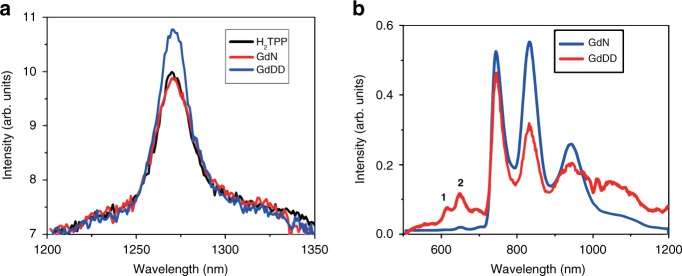
Emission spectra of the Gd(III) complexes. **a** The near-infrared ^1^O_2_ phosphorescence spectrum sensitized by GdDD, GdN, and the standard tetraphenylporphyrin H_2_TPP (in CHCl_3_. Absorbance = 0.05 at the excitation wavelength of 425 nm). **b** The 77 K phosphorescence spectrum of **GdDD** and **GdN** in MeOH (Conc.: 10 μM, *λ*_ex_ = 425 nm)

As a cross-system validation, the singlet oxygen quantum yield of **GdDD** was also examined in chloroform by comparison with the spectrum of the reference compound H_2_TPP (*Φ*_Δ_ = 55% in CHCl_3_). A new-generation anticancer agent **GdN**, which consists of only one porphyrin ring, with high-singlet oxygen quantum yield was selected to serve as a comparison. The near-infrared ^1^O_2_ phosphorescence spectra of **GdDD, GdN** and the reference are shown in Fig. 4a. From these spectra, the singlet oxygen quantum yields of **GdDD** and **GdN** were measured at 66% and 51%, respectively. The singlet-oxygen quantum yield was also evaluated in aqueous solution with a PBS buffer using rose bengal (RB) as the standard by absorption changes of the decomposition of 9,10-anthracenediyl-bis (methylene) dimalonic acid (ABDA) at 402 nm (Supplementary Figs. S15 and S16). The values of *Φ*_Δ_ were determined as 46% for **GdDD** and 42% for **GdN**. Hence **GdDD** displayed superior singlet oxygen generation in both organic and aqueous media. The comparison with two U.S. Food & Drug Administration approved PDT agents, porfimer sodium (Photofrin®) and 5-aminolevulinic acid (Levulan®) was made. Although **GdDD** shows lower-singlet oxygen quantum yield (46% in aqueous solution, Photofrin®: 89%; Levulan®: 56%), it has a much higher maximal absorptivity (**GdDD**: 223,872 M^−1^ cm^−1^ @412 nm, and 52480 M^−1^ cm^−1^ @580 nm) than these two commercial photosensitizers (Photofrin®: 3000 M^−1^ cm^−1^ @632 nm; Levulan®: 5000 M^−1^ cm^−1^ @632 nm)^44^. With a double-decker porphyrinato structure and the resulting high molar extinction coefficient values, **GdDD** shows great applicability in photodynamic effects, which is also consistent with the high brightness of **YbDD**. In vitro experiments have also been performed to practically compare photodynamic therapeutic efficiency in different cell lines, which also suggest **GdDD** as a potential PDT agent (Supplementary Figs. S25–S30 and Supplementary Table S4).

The energy gap between the antenna donor state and the lanthanide ion plays a crucial role in the energy transfer efficiency. The lowest triplet state of the lanthanide double-decker complex was determined experimentally from phosphorescence. The 77 K phosphorescence spectra of **GdDD** and **GdN** are shown in Fig. 4b. The zero-phonon lines are at a very similar wavelength ( ~ 745 nm: 13405 cm^−1^), and the prominent vibrational progression in the ring carbon–nitrogen stretching mode of 1410 cm^−1^ is at a lower energy. The triplet energy level is therefore located at 2610 cm^−1^ above the highest ^2^F_5/2_ level of Yb^3+^ in **YbDD**. The optimum energy gap has been given as between 2000 and 5000 cm^−1^ to eradicate back energy transfer^45,46^. The weak features marked 1 and 2 in Fig. 4b correspond to the singlet fluorescence bands S_1_(0,0) and S_1_(0,1), as in Fig. 3b for **YbDD** at 298 K. The triplet state lifetimes of **GdDD** and **GdN** at 77 K were measured as 0.21 ± 0.03 and 0.14 ± 0.02 ms, respectively.

### Absorption spectrum and transient absorption spectroscopy

Previous calculations of the energy levels of double-decker complexes have shown poor agreement with experiments^47,48^. Herein, the absorption spectrum was modeled from the optimized structure by two programs. First, an excited states calculation was performed using the RM1 semiempirical quantum chemistry method using the LUMPAC suite of programs^28,29^, and the calculated result is shown as the dashed blue line in Fig. 3a. The strong singlet-singlet transition is located at 346 nm. In the alternative calculation using ORCA^30^, this feature is shifted to lower energy at 530 nm (red dashed line, Fig. 3a).

**Fig. 5:**
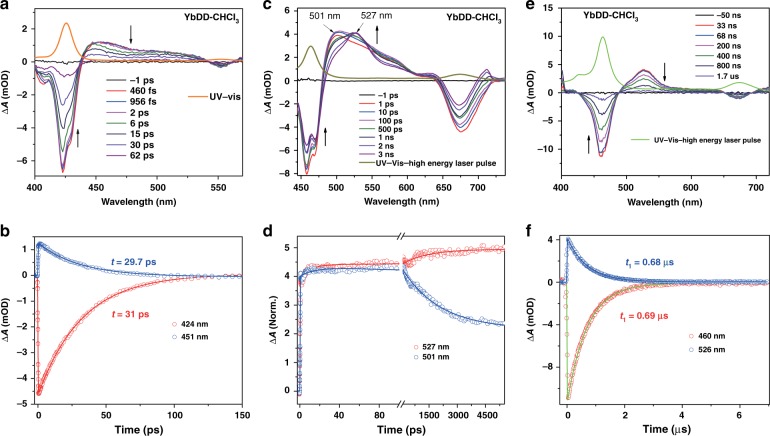
Transient absorption spectra of YbDD. **a** fs-TA spectra at different time delays at a low pump fluence (15 µJ cm^−2^), **b** ground state bleach recovery dynamics at 424 nm and excited state decay at 451 nm of **YbDD** in chloroform following 395 nm laser excitation. **c** Spectral evolution in fs-TA at 395 nm laser excitation having 45 µJ cm^−2^ fluence. **d** Normalized excited state decay kinetics at 501 and 527 nm. **e** ns-TA spectra at different time delays with a 45 µJ cm^−2^ pump fluence. **f** Ground state bleach recovery dynamics at 460 nm and the excited state decay at 526 nm of **YbDD** in chloroform following 395 nm laser excitation. The UV-vis spectra of **YbDD** are added in the upper panels to guide the ground state bleach

Transient absorption (TA) spectroscopy, as two-dimensional spectroscopy, was used to investigate both the spectral and temporal properties of the samples. The femtosecond (fs) TA spectra at different delay times for **YbDD** in chloroform at low laser fluence are displayed in Fig. 5a. The S_0_ → S_2_ Soret absorption band is shown in orange color, and its stimulated emission band has a small red shift with respect to the ground-state bleach and gives a negative signal^49^. The triplet–triplet (T_1_ → T_n_) absorption bands are observed at longer wavelengths (440–530 nm)^50^, with maximum intensity at 451 nm, corresponding to the terminal state energy of 35,578 cm^−1^. The lifetimes of the bleach and the excited state transients for **YbDD** were determined by monitoring at wavelengths of 424 and 451 nm, respectively. (Fig. 5b). The two results are effectively the same and are in the picosecond scale, denoting a rapid singlet-to-triplet intersystem crossing. The femtosecond TA absorption spectra were also obtained using a higher pump fluence (Fig. 5c). The pulsed laser with high fluence produces a thermal effect of the **YbDD**, which causes distortions of the porphyrin structures and results in significant redshifts in the electronic absorption spectra^51^. In contrast to the lower fluence, a redshift of the Soret band (Δ*λ* = + 36 nm) and the T_1_ → T_n_ absorption bands were observed. It is worth noting that the structural change was detected instantly by ultrafast TA spectroscopy: the peak at 501 nm started to shift to 527 nm after 100 ps, and the whole conformation changing process was completed in nanoseconds (Fig. 1d). Furthermore, the formation of the triplet state from the singlet excited state is clearly observed from the kinetics at 527 nm in Fig. 5d. In contrast to the 527 nm, the excited singlet state at 501 nm de-excited exponentially to the ground states. However, when using nanosecond (ns) TA spectroscopy, the detailed kinetics of the triplet–triplet absorption peak at approximately 526 nm could not be resolved since the conformation was changing too quickly (Fig. 5e). The ground state bleach recovery lifetime was extracted from the nanosecond TA spectra (0.69 μs), which is consistent with the decay lifetime by monitoring deactivation of the triplet state signal at 526 nm (0.69 μs) (Fig. 5f). Isosbestic points were found in all TA spectra (Fig. 5a, c, e), which suggest that only one single photoexcited species was formed in each case.

## Discussion

The porphyrin moiety acts as a viable antenna under excitation at 425 nm for the ytterbium ion. Absorption by the Soret band is followed by an internal conversion cascade to lower singlets and intersystem crossing to T_1_. Calculation shows that there is considerably more than one singlet and one triplet state involved in this cascade. The energy transfer from T_1_ lifts the Yb^3+^ ion from the ^2^F_7/2_ ground state to ^2^F_5/2_. This change of Δ*J* = 1 is consistent with the first order selection rules for exchange or quadrupole interaction. On the other hand, the donor T_1_ → S_0_ nonradiative transition is dipole forbidden. Considering the separation between Yb^3+^ and the porphyrin ring of less than 2 Å, the dominant energy transfer mechanism is most likely to be the exchange mechanism.

We report here the first water-soluble lanthanide–porphyrin double-decker complex with structural characterization using NMR, HRMS, STM, and computational chemistry techniques. NIR imaging and cytotoxic ^1^O_2_ generation of the complexes have also been developed. In our work, the major improvement for bioapplications—considering the low-quantum yield of Yb^3+^ in the NIR region—comes from the enhancement of the brightness of the potential bioimaging probe **YbDD**. This property, brightness, has not yet been widely recognized as the yardstick for applicability, compared with quantum yield, but it shows higher practical significance for bioimaging purposes.

## Materials and methods

### General synthesis

Dichloromethane (DCM), methanol (MeOH) and n-hexanol were dried by refluxing with calcium hydride (CaH_2_) before setting up a reaction. All the chemicals and reagents were of high quality and could be used directly. Reaction processes were monitored by thin-layer chromatography, and further monitored using a UV lamp. Silica gel or Al_2_O_3_ were used for purification in most cases. High-performance liquid chromatography (HPLC) methods were used for final products with high polarity. NMR spectra were recorded by either a 400 (^1^H: 400 MHz, ^13^C: 100 MHz) or a 500 (^1^H: 500 MHz, ^13^C: 1250 MHz) spectrometer. High-resolution mass spectra were recorded on a Bruker Autoflex II (Bruker Dalton GmBH) MALDI-TOF mass spectrometer (characterized by *m/z*).

### Procedures for the preparation of 5,15-bis(3-(2-(2-methoxyethoxy)ethoxy)phenyl)porphyrin (Por-2DEG)

Di(1H-pyrrol-2-yl)methane (788.84 mg, 5.4 mmol) was dissolved in 1 L dry DCM in a round flask, and 3-(2-(2-methoxyethoxy)ethoxy)benzaldehyde (1.21 g, 5.4 mmol) was added to the solution which was stirred for 30 min under a nitrogen atmosphere to remove oxygen. Next, trifluoroacetic acid (0.24 mL, 3.24 mmol) was added slowly. The mixture was stirred at room temperature for 3 h under a nitrogen atmosphere. After this time, 2,3-dichloro-5,6-dicyano-1,4-benzoquinone (DDQ) (1.47 g, 6.48 mmol) was added, and the mixture was stirred for an additional 1 h. Then, 2 mL of triethylamine was added to quench the unreacted TFA. The mixture was stirred for 10 min, and the solvent was removed. The product was purified through silica gel with the solvent gradient DCM: MeOH (100:1).

### General procedures for the preparation of LnDD (Ln = Yb, Er, Gd and La)

5,15-bis(3-(2-(2-methoxyethoxy)ethoxy)phenyl)porphyrin (80.0 mg, 0.12 mmol), Ln(acac)_3_.*x*H_2_O (0.48 mmol), and 1,8-diazabicyclo(5.4.0)undec-7-ene (DBU, 114 μL, 0.79 mmol) were dissolved in 10 mL dry hexanol. The mixture was bubbled with nitrogen for 20 min at room temperature and then refluxed for 12 h under a nitrogen atmosphere. After reaction completion, the contents were cooled down to room temperature and mixed with 30 mL hexane. The precipitate was dissolved in DCM and transferred to an Al_2_O_3_ column for purification. (DCM: MeOH 20:1). HPLC was then used for further purification with a preparative column (C18, 10.0 × 250 mm, 5 μm particle size). The final product was confirmed by MALDI-TOF mass spectral analysis operating in the positive-ion mode using the α-cyano-4-hydroxycinnamic acid matrix.

### Scanning tunneling microscopy

An HOPG sample (10 × 10 mm) (SPI) was mounted on a Ta plate. The surface was exfoliated with scotch tape and the surface was verified in a UHV Omicron VT-STM at 10–9 mbar. The STM tip (VT-STM Omicron) was Pt/Ir and was prepared by degassing at 100 °C for 10 h and then further cleaned by electron bombardment using a tip preparation tool (Omicron), 2 A, 2 mA, and 950 V for 2 s. In-plane *x*–*y* calibration was performed by measuring the atomically resolved HOPG surface lattice parameters. To prepare the monolayer film samples, a droplet (5–25 μL) (1 mmol) of a solution in chloroform or toluene was placed in the centre of a clean surface of HOPG and allowed to evaporate at room temperature. The films were dried under roughing vacuum at 10^−2^ mbar for 2 h then transferred to the UHV system for STM imaging.

### General spectroscopic characterizations

The absorption spectra of the final products were measured in aqueous solution in the range 200–800 nm using an HP Agilent UV-8453 Spectrophotometer. The emission spectra from 400 to 1600 nm were obtained by the Fluorolog-3 TCSPC (Horiba) combined fluorescence lifetime and steady-state spectrometer. The spectrometer was equipped with an NL-C2 Pulsed Diode Controller NanoLED, which produces picosecond and nanosecond optical pulses at a wide range of wavelengths from the ultraviolet to NIR.

### TA spectroscopy

Helios spectrometers (Ultrafast systems, FL, USA) were used to perform femtosecond transient absorption spectroscopy. The detailed experimental setup of the fs-TA is given in the literature^52^. Briefly, a white-light continuum probe pulse was generated in a 2-mm-thick sapphire plate utilizing a small fraction of the fundamental output of a Ti:sapphire femtosecond regenerative amplifier that was operating at 800 nm with 35 fs pulses and a repetition rate of 1 kHz. Pump pulses at 395 nm were formed in an optical parametric amplifier (Newport Spectra-Physics). In a 2-mm-thick cuvette cell containing the sample solutions, the pump and probe pulses were overlapped temporally and spatially. The probe light transmitted from the sample was gathered and focused on a broadband UV–visible detector to observe the change in absorbance (Δ*A*). The nanosecond TA spectroscopic measurements were also performed at 395 nm following laser pulse excitation. The ns-TA spectra were recorded using the pump-probe EOS setup (Ultrafast systems, FL, USA), in which a standard probe beam was split into two: one travels through the sample, and the other one is sent directly to the reference spectrometer, which monitors the fluctuations in the probe beam intensity. The detailed experimental setup of the EOS can be found elsewhere^53^.

## Supplementary information

SUPPLEMENTAL INFORMATION for Impressive Near-Infrared Brightness and Singlet Oxygen Generation from Strategic Lanthanide–Porphyrin Double–Decker Complexes in Aqueous Solution
