# Supplementary material for: Impressive near-infrared brightness and singlet oxygen generation from strategic lanthanide–porphyrin double-decker complexes in aqueous solution
Source: Light Sci Appl. 2019 May 22;8:46. doi: 10.1038/s41377-019-0155-9 (PMC6529462; doi:10.1038/s41377-019-0155-9)
Supplement: Supplementary file 1 — SUPPLEMENTAL INFORMATION for Impressive Near-Infrared Brightness and Singlet Oxygen Generation from Strategic Lanthanide–Porphyrin Double–Decker Complexes in Aqueous Solution [file 41377_2019_155_MOESM1_ESM.docx]

**SUPPLEMENTAL INFORMATION**

**for**

**Impressive Near-Infrared Brightness and Singlet Oxygen Generation from Strategic Lanthanide–Porphyrin Double–Decker Complexes in Aqueous Solution**

Jingxiang Zhang^1,4,#^, Wai-Lun Chan^1,#^, Chen Xie^1,#^, Yan Zhou^1^, Ho-Fai Chau^2^, Partha Maity^3^, George T. Harrison^3^, Aram Amassian^3^, Omar Mohammed^3^, Peter A. Tanner^*,1^, Wai-Kwok Wong^*,1^, and Ka-Leung Wong^*,1^

^1^Department of Chemistry, Hong Kong Baptist University, Kowloon Tong, Hong Kong S.A.R., China, [klwong@hkbu.edu.hk](mailto:klwong@hkbu.edu.hk)

^2^Department of Biology, Hong Kong Baptist University, Kowloon Tong, Hong Kong S.A.R., China

^3^KAUST Solar Center, Division of Physical Science and Engineering, King Abdullah University of Science and Technology (KAUST), Thuwal 23955-6900, Kingdom of Saudi Arabia

^4^Hanshan Normal University, Chaozhou, Guangdong Province, China

^#^These authors contributed equally to this work.

**Contents**

**1.Experimental**

**1.1 Scheme S1. The synthetic scheme of double-decker porphyrinate**

**lanthanide complexes (LnDD)**

**1.2 Characterization of Por-2DEG**

**1.3 Characterization of LnDD**

**1.4 HPLC characterization of LnDD**

**Table S1 Solvent gradient used for preparative HPLC**

**Figure S1. HPLC chromatograms of LnDD**

**1.5 Methodology of scanning tunneling microscopy**

**1.6 Mass spectroscopy characterizations**

**Figure S2. Mass spectrum (MALDI-TOF) of Por-2DEG**

**Figure S3. Mass spectrum (MALDI-TOF) of YbDD**

**Figure S4. Mass spectrum (MALDI-TOF) of ErDD**

**Figure S5. Mass spectrum (MALDI-TOF) of GdDD**

**Figure S6. Mass spectrum (MALDI-TOF) of LaDD.**

**1.7 NMR characterizations**

**Figure S7. 400 MHz-1H-NMR (CDCl_3_) spectrum of Por-2DEG**

**Figure S8. HNMR spectrum of LaDD in CDCl_3_:DMSO-*d6***

**Figure S9. Variable-temperature NMR spectra of LaDD in CD_2_Cl_2_**

**1.8 Stability tests**

**Figure S10. Emission spectra of YbDD in different pH/concentrated PBS buffers**

**2. Photophysical measurements**

**2.1 General photophysical measurements**

**2.2 NIR emission and quantum yield**

**Figure S11. The NIR emission spectra of YbDD in DCM and water**

**Figure S12. Comparison of NIR emission spectra of Yb-complexes**

**Figure S13. The NIR emission spectra of Yb-complex in toluene and MeOH**

**Table S2 The NIR emission quantum yields of YbDD in various solvents**

- 1. **Photostability tests**

**Figure S14.** **Absorption spectra of YbDD aqueous solutions (with/without covering) before and after light irradiation.**

- 1. **Methodology of transient absorption spectroscopy**
  2. **Singlet oxygen quantum yield measurements**

**2.5.1 Singlet oxygen quantum yield in organic solvent (CHCl_3_) by emission**

**2.5.2 Singlet oxygen quantum yield in PBS buffer by absorption**

**Figure S15. Time-dependent absorption changes of ABDA**

**Figure S16. Plots of the changes in absorbance of ABDA at 402 nm**

**2.6 Lifetime decay measurements**

**Figure S17. NIR emission decay of YbDD in water**

**Figure S18. NIR emission decay of YbN in water**

**2.7 Low temperature (77 K) emission spectra measurement**

**Figure S19. The 77 K emission spectra of YbDD and ErDD in MeOH**

**Figure S20. The 298 K absorption spectra of LnDD in aqueous solution**

**Figure S21.The visible and NIR emission spectra of YbDD in aqueous solutio**

**Figure S22. The visible and NIR emission spectra of ErDD in aqueous solution**

**Figure S23. The emission spectrum of GdDD in aqueous solution**

**Figure S24. The emission spectrum of LaDD in aqueous solution**

**Table S3. Photophysical properties of LnDD**

**3. Photodynamic therapy studies**

**Figure 25. Dark cytotoxicity of GdDD and ErDD in HeLa cells**

**Figure 26. Light cytotoxicity of GdDD and ErDD in HeLa cells**

**Figure 27. Dark cytotoxicity of GdDD and ErDD in MRC5 cells**

**Figure 28. Light cytotoxicity of GdDD and ErDD in MRC5 cells**

**Figure 29. Fluorescence Intensity of emission in normaxia and hypoxia conditions.**

**Figure 30. Fluorescence spectra of Singlet Oxygen Sensor Green of different samples in normaxia and hypoxia conditions.**

**Table S4. Photo and Dark cytotoxicity of GdDD, ErDD, and GdN**

**4. Calculations**

**Figure S31. Optimized structure of the porphyrin dianion**

**Figure S32a/b. Optimized structure of YbDD using the RM1 model in LUMPAC**

**Figure S33. Optimized structure of YbDD using ORCA with the BP86 functional**

**Figure S34. LUMO and HOMO of YbDD**

**Figure S35. Optimized structure in Firefly of YbDD using the PBE0 functional**

**Figure S36. Optimized structure in ORCA of AlDD using the BP86 functional**

**Figure S37. Optimized structure in ORCA of [YbDD]^-^ using the PBeh-3c functional**

**References**

**1. Experimental**

**
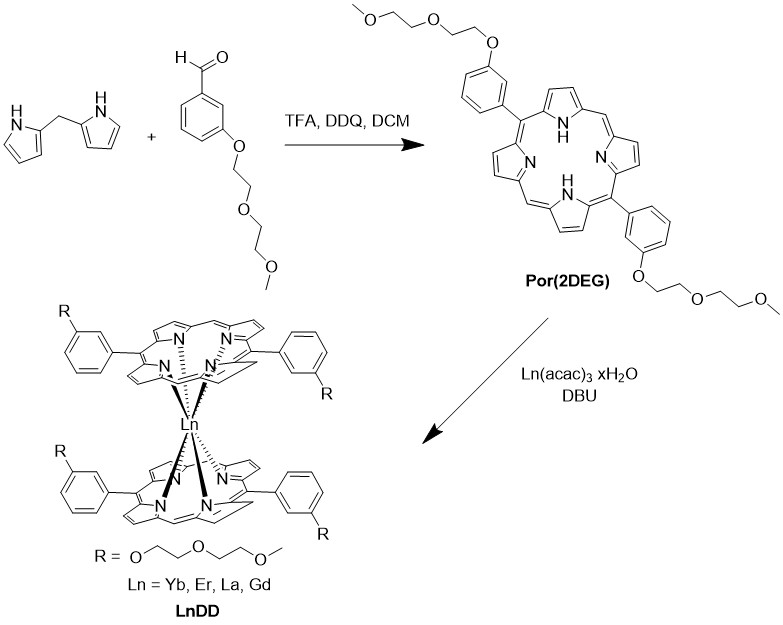
**

**1.1 Scheme S1.** The synthetic scheme of the double decker porphyrinate lanthanide complexes.

**Syntheses of LnDD (Ln = Yb, Er, Gd and La)**

The solvents used were dried before setting up a reaction. Dichloromethane (DCM), methanol (CH_3_OH) and n-hexanol were dried by refluxing with calcium hydride (CaH_2_). All the chemicals and reagents were of high quality and could be used directly. Reaction processes were monitored by TLC (thin-layer chromatography), and further monitored by a UV lamp. Silica gel or Al_2_O_3_ were used for purification in most of the cases. For final products with high polarity, HPLC methods were used. NMR spectra were obtained by either a 400 (^1^H: 400 MHz, ^13^C: 100 MHz) or a 500 (^1^H: 500 MHz, ^13^C: 1250 MHz) spectrometer. High-resolution mass spectra were recorded on a Bruker Autoflex MALDI - TOF mass spectrometer (characterized by *m/z*).

**Preparation of 5,15-bis(3-(2-(2-methoxyethoxy)ethoxy)phenyl)porphyrin (Por-2DEG)**

Di(1H-pyrrol-2-yl)methane (788.84 mg,5.4 mmol) was dissolved in 1 L dry DCM in a round flask and 3-(2-(2-methoxyethoxy)ethoxy)benzaldehyde (1.21 g, 5.4 mmol) was added to the solution which was stirred for 30 min under a nitrogen atmosphere to remove oxygen. Then Trifluoroacetic acid (TFA) (0.24 mL, 3.24 mmol) was added slowly. The mixture was stirred at room temperature for 3 h under a nitrogen atmosphere. After this time, 2,3-Dichloro-5,6-dicyano-1,4-benzoquinone (DDQ) (1.47 g, 6.48 mmol) was added and the mixture was stirred for a further 1 h. Then 2 mL Triethylamine (TEA) was added to quench the unreacted TFA. The mixture was stirred for 10 min, and the solvent was removed. The product was purified through silica gel with the solvent gradient DCM: MeOH(100:1).

**Preparation of LnDD (Ln = Yb, Er, Gd and La)**

5,15-bis(3-(2-(2-methoxyethoxy)ethoxy)phenyl)porphyrin (80.0 mg, 0.12 mmol), Yb(acac)_3_.*x*H_2_O (0.48 mmol), 1,8-Diazabicyclo[5.4.0]undec-7-ene (DBU) (114 μL, 0.79 mmol) were dissolved in 10 mL dry hexanol. The mixture was bubbled with nitrogen for 20 min at room temperature and then refluxed for 12 h under a nitrogen atmosphere. After completion, it was cooled down to room temperature and then mixed with 30 mL Hexane. The solid was dissolved in DCM and transferred to an Al_2_O_3_ column for purification. (DCM: MeOH 20:1, the second point in TLC). HPLC was then used for further purification with a preparative column (C18, 10.0 × 250 mm, 5 μm particle size). The final product was confirmed by MALDI-TOF mass spectral analysis. (Autoflex Ⅱ TOF/TOF mass spectrometer Bruker Dalton GmBH) operating in the positive ion mode using the α-cyano-4-hydroxycinnamic acid (CHCA) matrix.

**1.2 Characterization of 5,15-bis(3-(2-(2-methoxyethoxy)ethoxy)phenyl)porphyrin (Por-2DEG)**

**Por-2DEG**: ^1^HNMR (CDCl_3_): -3.16 (s, 2H), 0.387 (s, 9H), 3.37 (s, 6H), 3.60 (d, 4H), 3.77 (d, 4H), 3.96 (d, 4H), 4.37 (d, 4H), 7.30 (d, 2H), 7.60 (d, 2H), 7.87 (d, 4H), 9.10 (d, 2H), 9.37 (d, 2H), 10.30 (s, 2H), MS (MALDI) calcd. for C_42_H_42_N_4_O_6_ [M+H]^+^ 699.3123, found. 699.3173.

**1.3 Charaterization of LnDD (Ln = Yb, Er, Gd and La)**

**YbDD:** Yield: 45%; MALDI-TOF MS: calcd. for C_84_H_80_N_8_O_12_Yb [M]^+^ 1566.5312, found: 1566.5379. HPLC characterization: retention time = 15.518 min.

**ErDD**: The same procedure as for YbDD, replacing Yb(acac)_3_.*x*H_2_O with Er(acac)_3_.*x* H_2_O; Yield: 45%. MALDI-TOF MS: calcd. for C_84_H_80_N_8_O_12_Er [M+H]^+^ 1561.5214, found: 1561.5352. HPLC characterization: retention time = 15.585 min.

**GdDD**: The same procedure as for YbDD, replacing Yb(acac)_3_.*x*H_2_O with Gd(acac)_3_.*x* H_2_O; Yield: 10%. MALDI-TOF MS: calcd. for C_84_H_80_N_8_O_12_Gd [M+H] ^+^ 1551.5229, found: 1551.5362. HPLC characterization: retention time = 15.495 min.

**LaDD**: The same procedure as for YbDD, replacing Yb(acac)_3_.*x*H_2_O with La(acac)_3_.*x* H_2_O; Yield: 10%. ^1^HNMR (CDCl_3_): 1.879(s, 12H), 2.883(m, 8H), 3.051 (m, 8H d), 3.260(d, 8H), 3.685(s, 8H), 6.730 (m, 4H), 7.060 (m, 4H), 7.160 (M, 8H)), 8.435 (d, 8H), 8.814 (d, 8H), 9.747 (s, 4H). MALDI-TOF MS: calcd. for C_84_H_80_N_8_O_12_La [M+H]^+^ 1532.5032, found: 1532.4982. HPLC characterization: retention time = 15.098 min.

**1.4 HPLC characterization of LnDD (Ln = Yb, Er, Gd and La).**

**Table S1. Solvent gradient used for preparative HPLC.**

| Time /min | 0.05% HCOOH in Water /% | MeOH /% |
| --- | --- | --- |
| 0 | 30 | 70 |
| 5 | 20 | 80 |
| 15 | 0 | 100 |
| 20 | 0 | 100 |
| 30 | 20 | 80 |
| 35 | 30 | 70 |

**
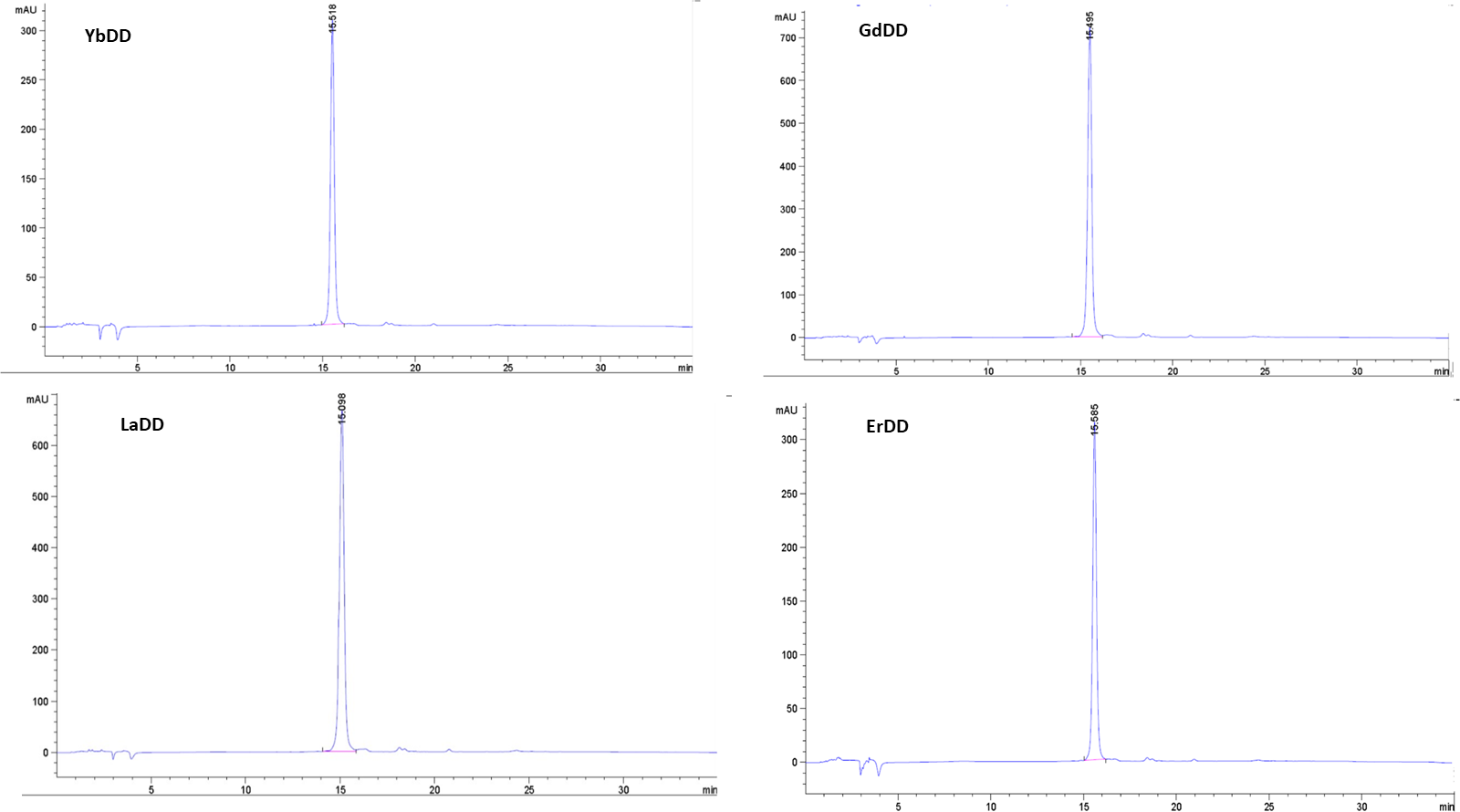
**

**Figure S1.** HPLC chromatograms of **LnDD** (Ln = Yb, Er, La and Gd). Elution conditions: column, Agilent ZORBAXSB-C18 (4.6 × 150 mm, particle size 5; flow rate, 1.0 mL min^-1^; gradient elution; detection wavelength, 430 nm. Retention time: **LaDD** 15.098**, YbDD** 15.518 min**, GdDD** 15.495 min**. ErDD** 15.585 min.

**1.5 Methodology of scanning tunneling miscroscopy**

Highly Orientated Pyrolytic Graphite HOPG (0001) single crystals were cleaned by cleaving in air using scotch tape. A drop (15 µL) of **YbDD** (10^-6^ -10^-5^ M) dissolved in chloroform was placed on the surface and allowed to dry at RT in ambient conditions, depositing the film. The sample was dried at 10^-2^ mbar (2 hours) then transferred to the UHV chamber for STM analysis.

STM was performed at room temperature using an Omicron multi-probe (XPS/STM) UHV (10^-9^ mbar) system, which housed a VT-STM. Purchased STM tips (Pt/Ir) (Omicron) were prepared by repeated exposure *in-situ* to electron bombardment (950 V, 2 mA, 2 s) with a tip preparation tool (Omicron). STM tips were judged to be suitable for measurements when the surface of clean HOPG(0001) could be atomically resolved. In plane x-y distance calibration was performed from measurements of clean HOPG(0001). During measurements STM tips were conditioned by +5 V sample bias, 1.6 ms, open feed-back loop pulses.

**1.6 Mass spectroscopy characterizations**

**
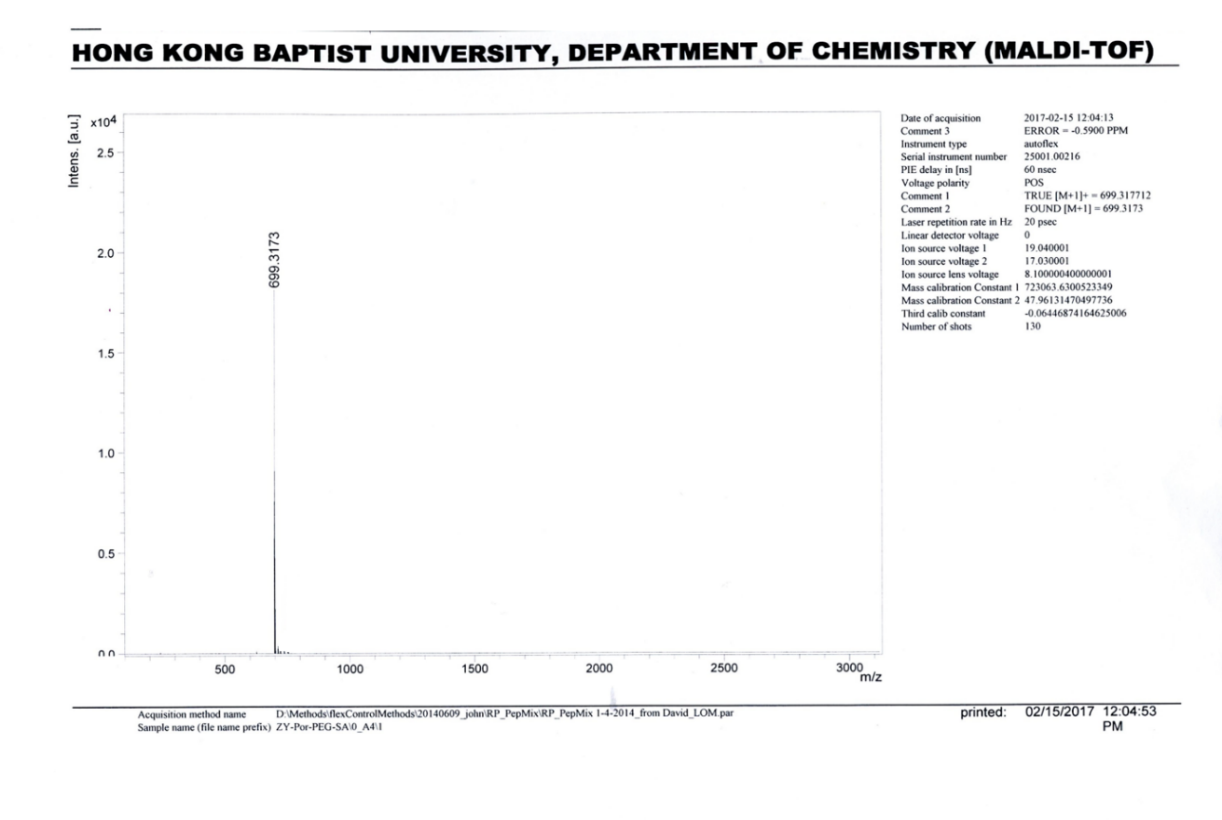
**

**Figure S2.** Mass spectrum **(**MALDI-TOF) of Por-2DEG.


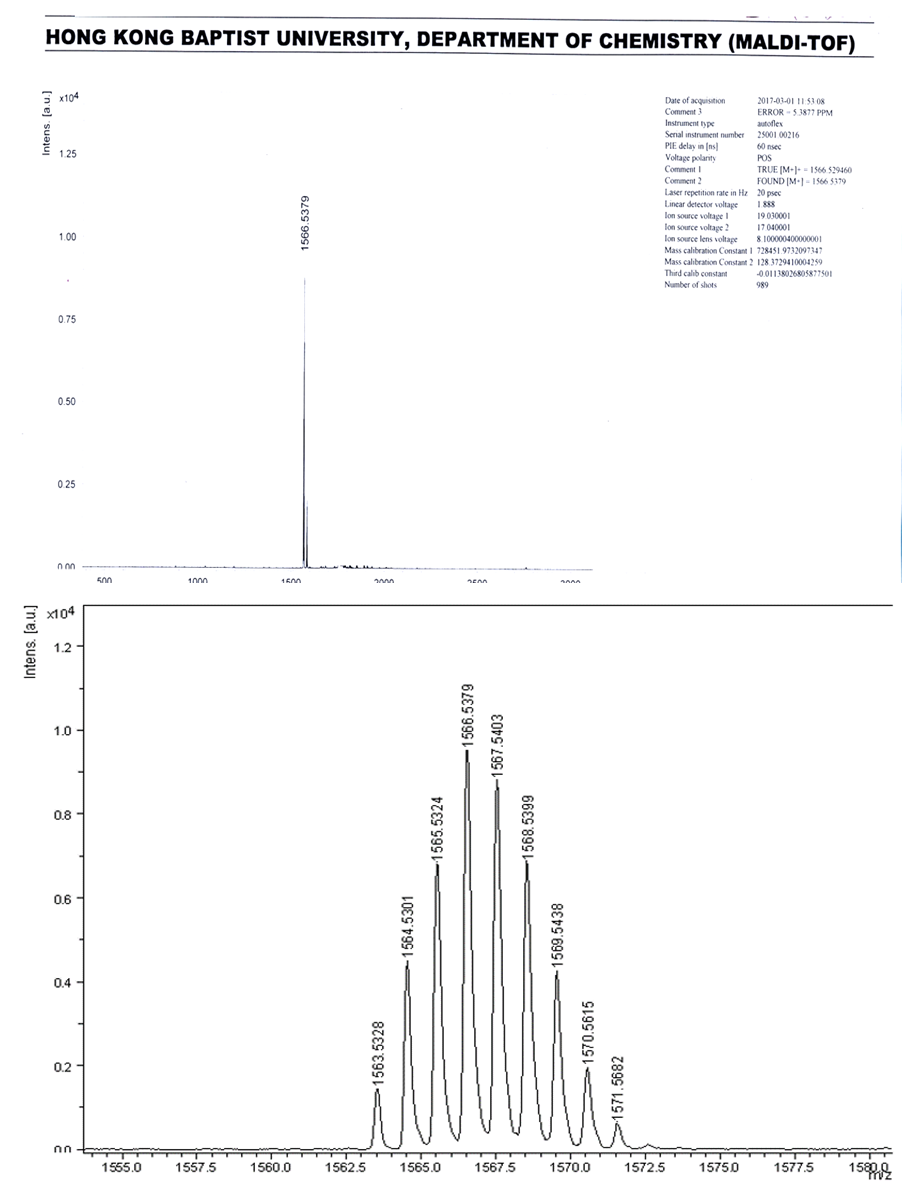


**Figure S3.** Mass spectrum **(**MALDI-TOF) of **YbDD.**


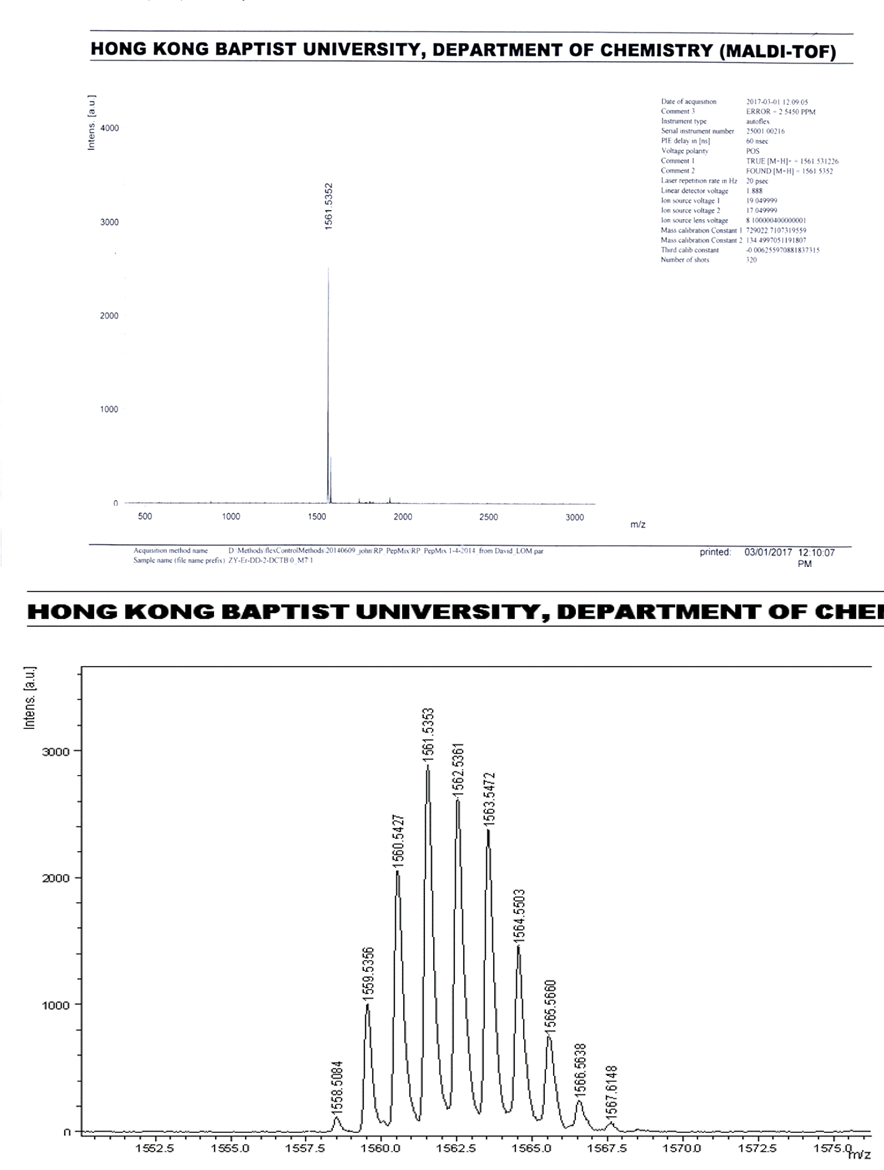


**Figure S4.** Mass spectrum **(**MALDI-TOF) of **ErDD.**


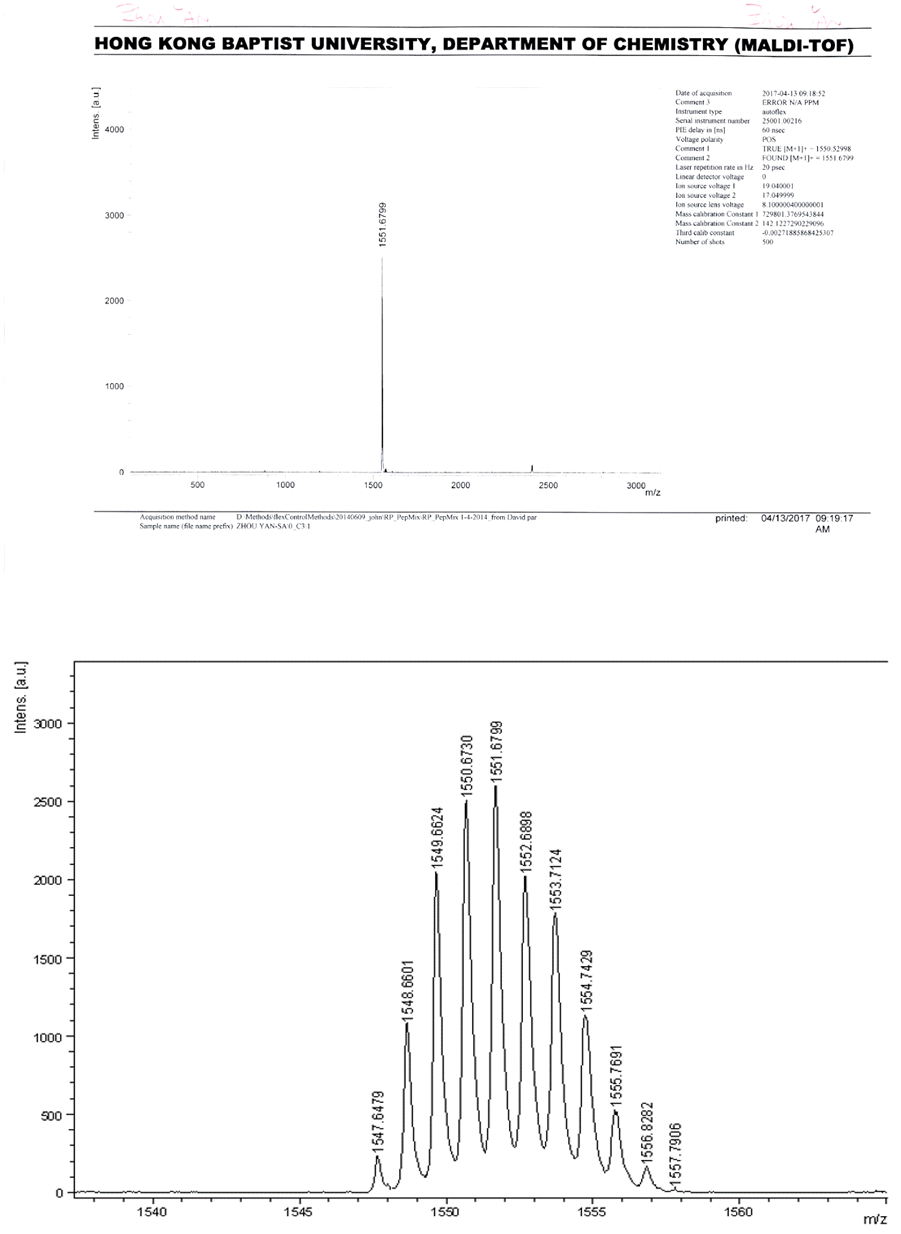


**Figure S5.** Mass spectrum **(**MALDI-TOF) of **GdDD.**


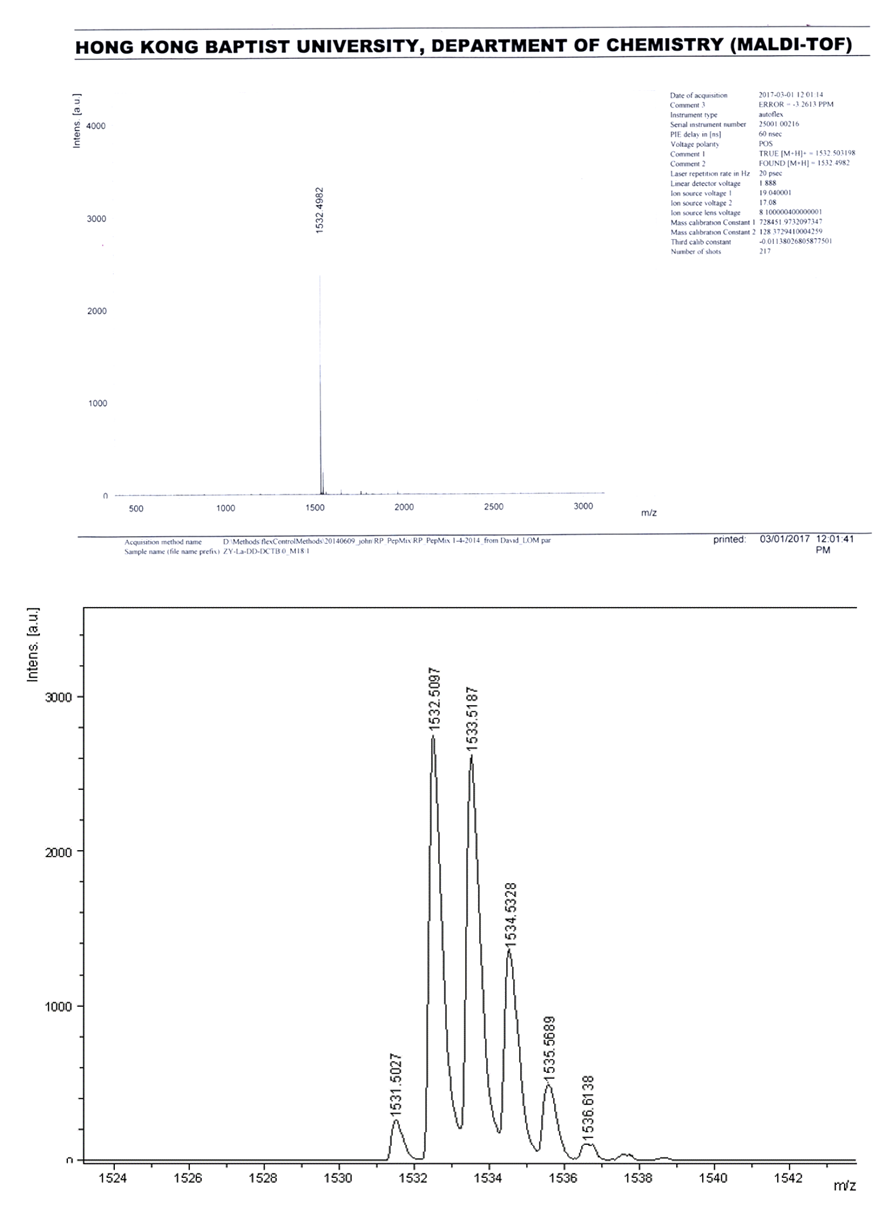


**Figure S6.** Mass spectrum **(**MALDI-TOF) of **LaDD**.

**1.7 NMR characterizations**

**
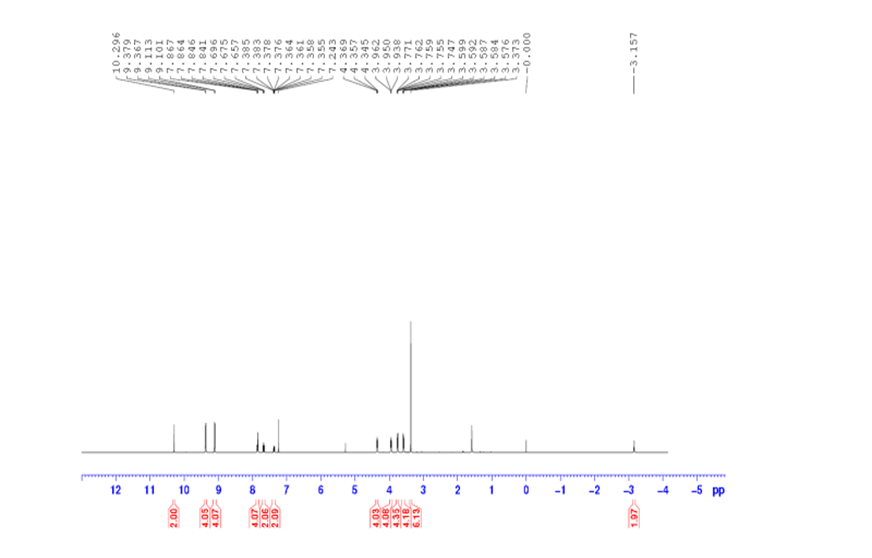
**

**Figure S7.** 400 MHz-^1^H-NMR (CDCl_3_) spectrum of Por-2DEG


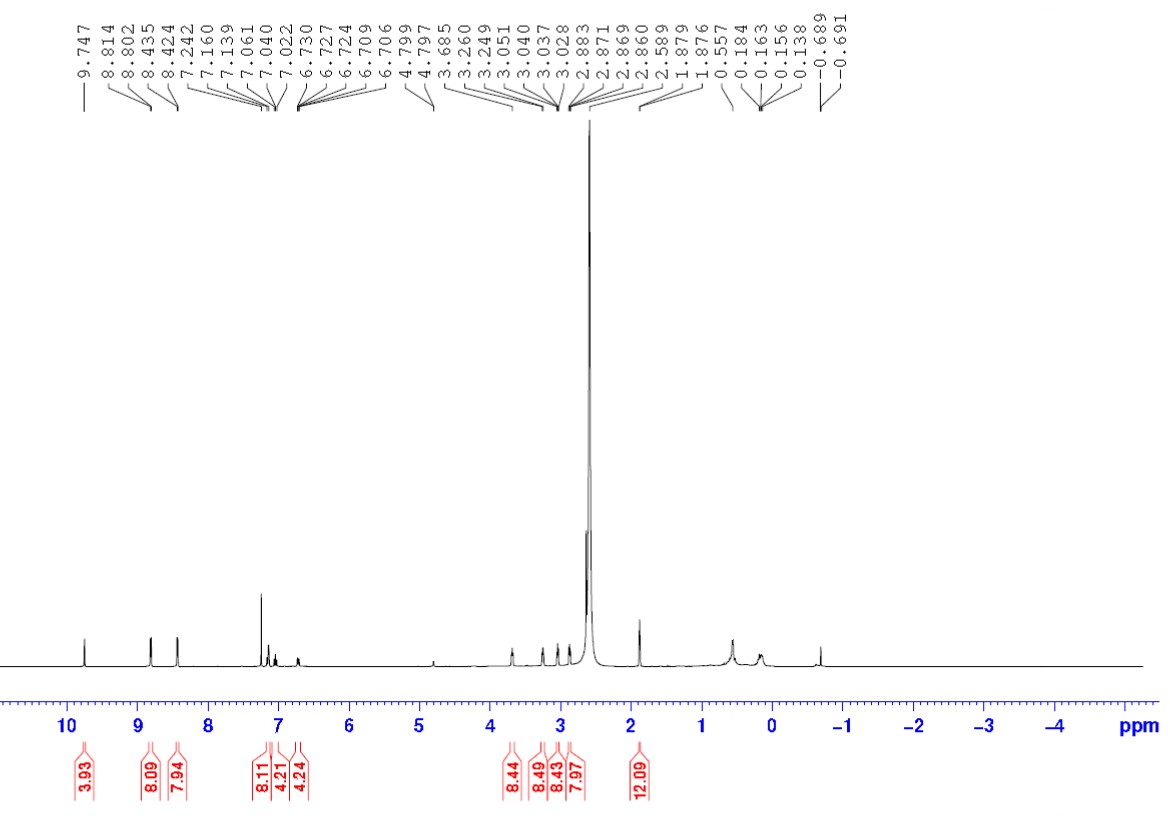


**Figure S8.** NMR spectrum of **LaDD** in CDCl_3_:DMSO-*d6* 1:1 mixed with 1% hydrazine hydrate.


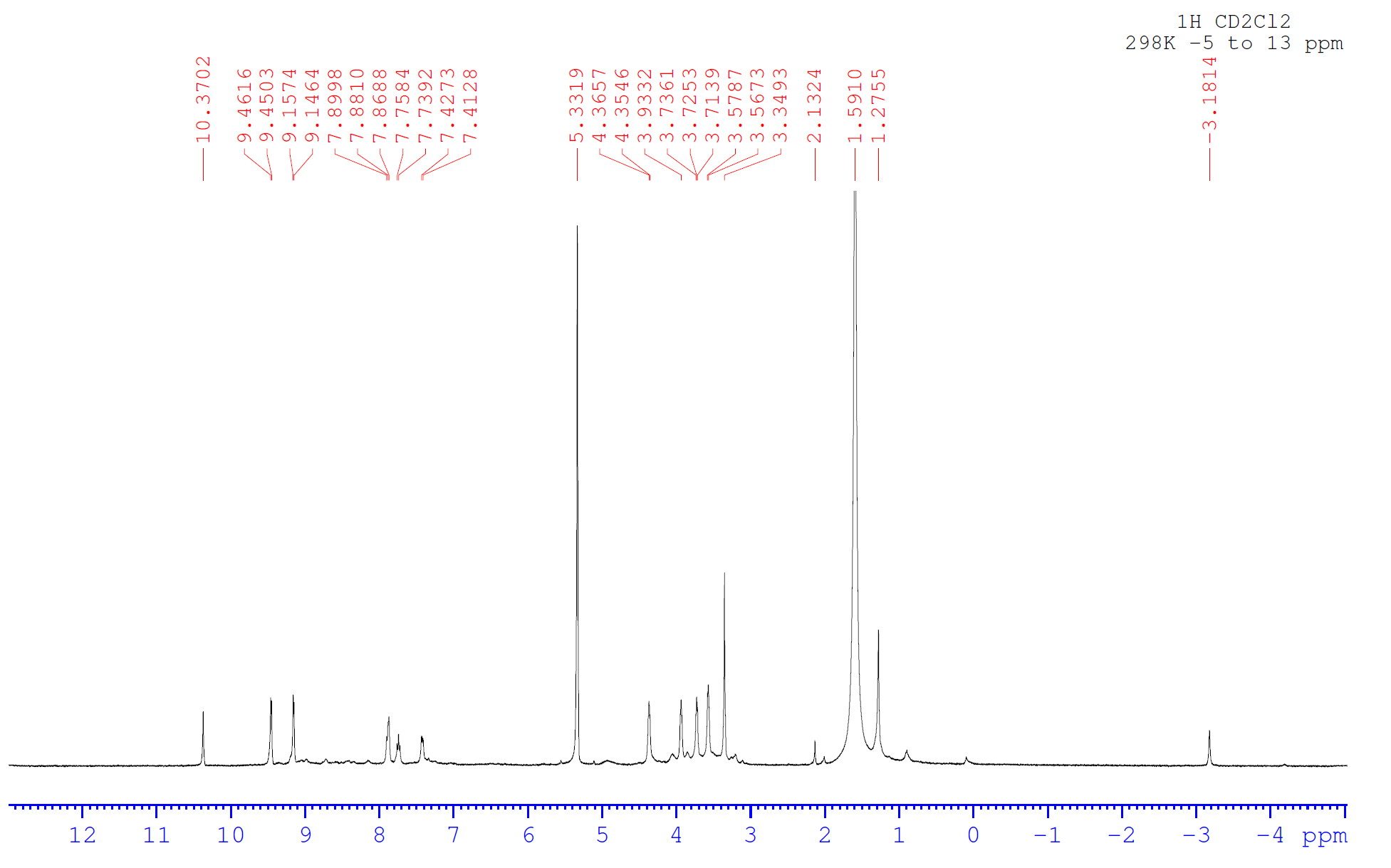


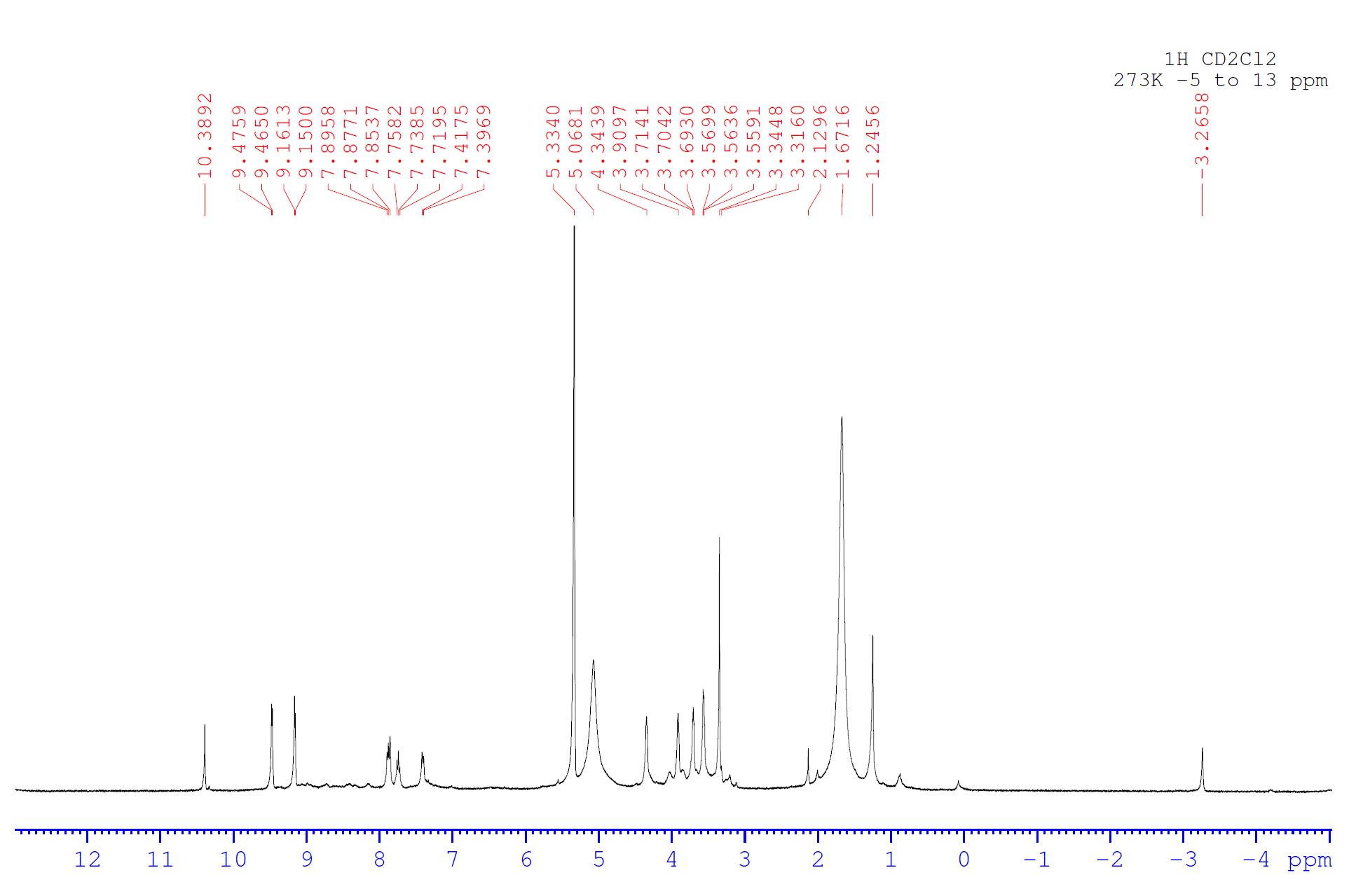


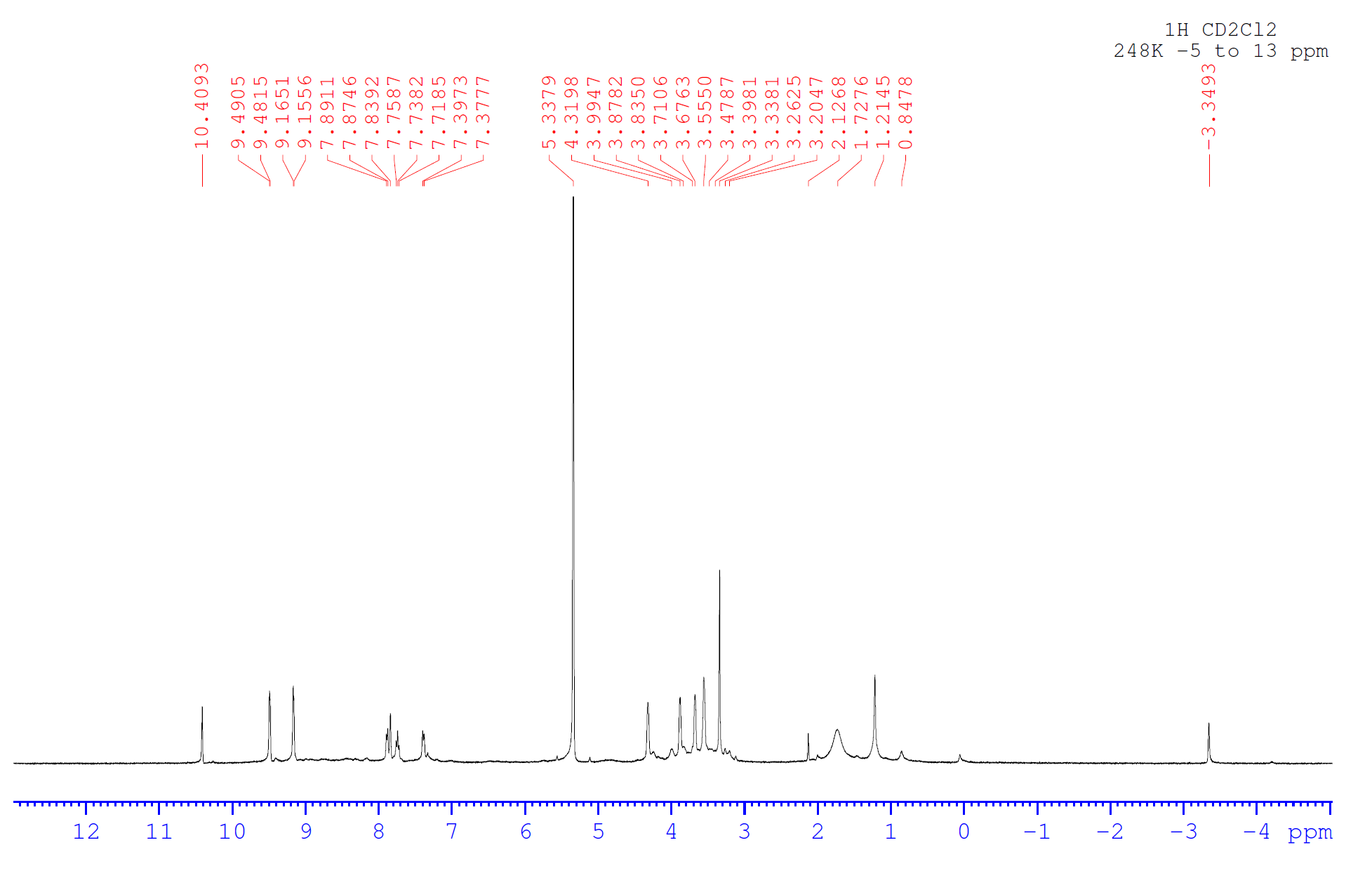


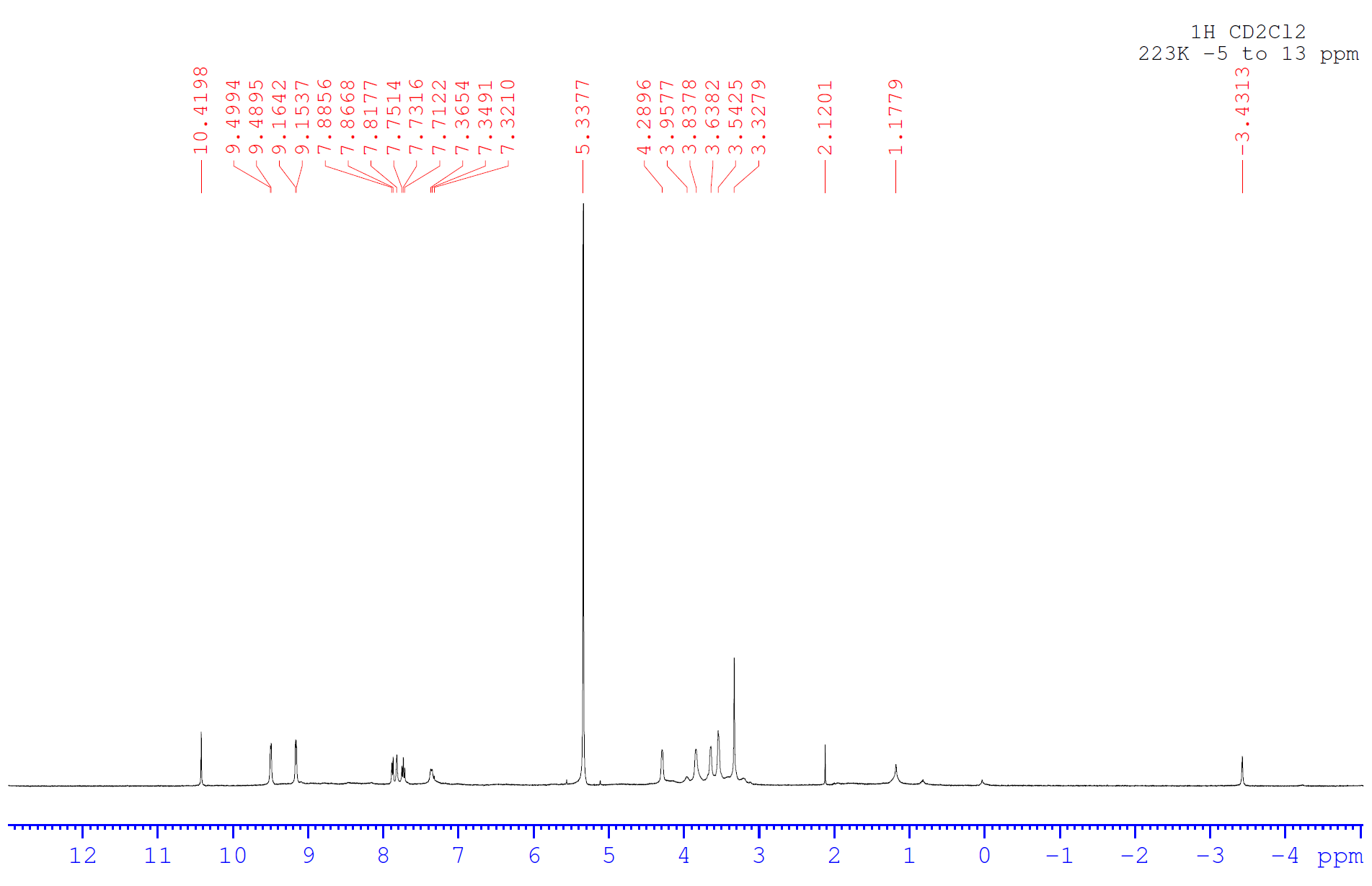


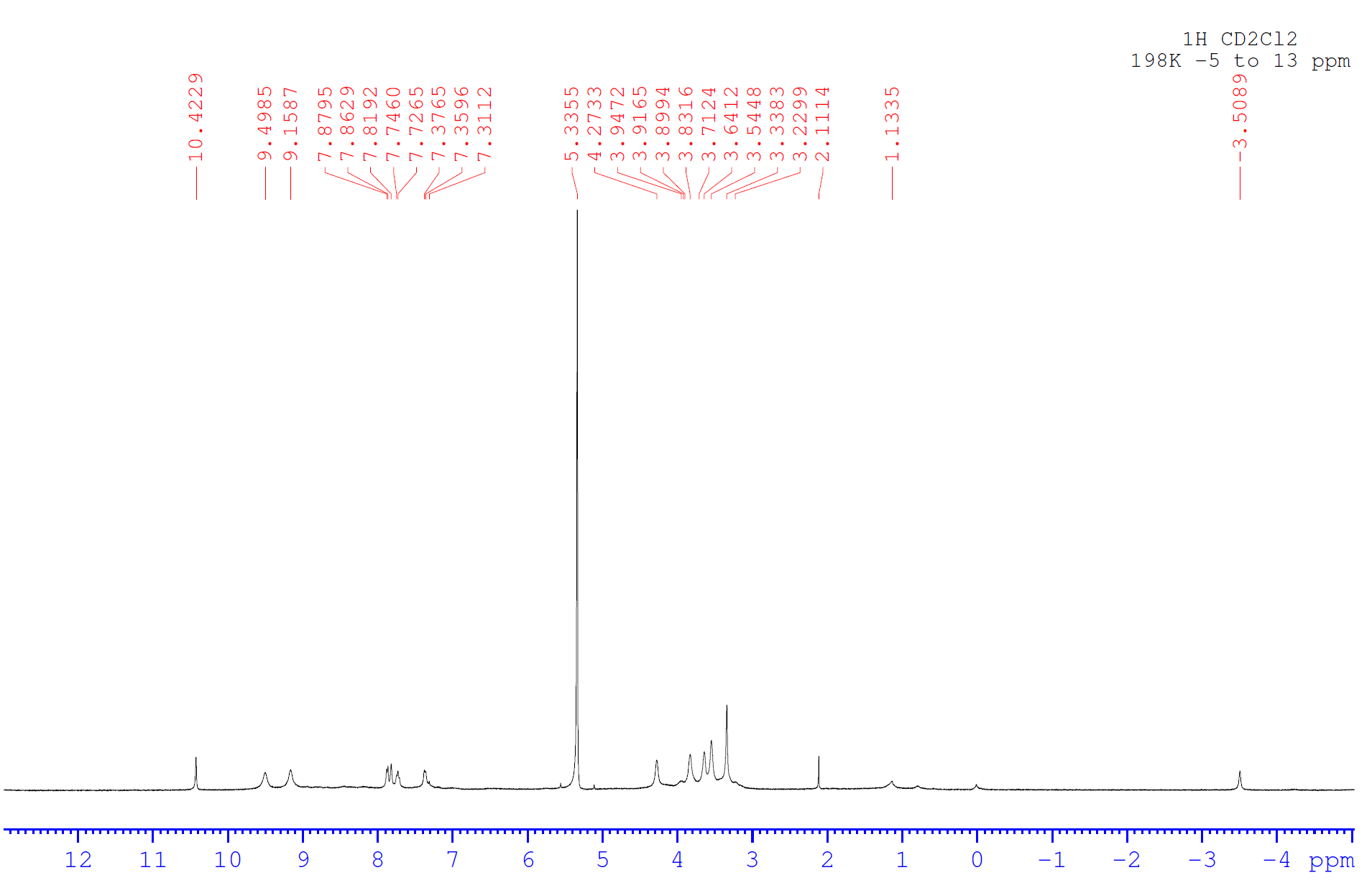


**Figure S9.** Variable-temperature NMR spectra of **LaDD** in CD_2_Cl_2_ (temp. from top: 298K, 273K, 248K, 223K, 198K).

Variable-temperature (VT) NMR experiments have been performed to investigate any tautomerism in the structures. According to VT NMR results, NH proton was more shielded (moving from -3.18 to -3.51 ppm) when the temperature decreased from 298 K to 198 K, which could result from less motion and more planarity of the porphyrin rings at low temperature. However, there is no observable change for the β-pyrrolic proton signals (doublet-doublet peaks, 9~10 ppm), which means that tautomerization of the one free proton is unlikely.

**1.8 Stability tests**

**Figure S10.** Emission spectra of 100 µM **YbDD** in 1X or 5X concentrated Phosphate-buffered saline at pH=5 or pH=7.

The emission profiles do not have dramatic changes for **YbDD** in different solvents, and typical f-f transition emission peaks are observed clearly. This proves the stability of YbDD in simulated tumor microenvironments (pH 5~7, similar osmolarity and ion concentrations matching with human body).

Considering the most intense emission peak at 975 nm belong to Yb(III), at the same concentration of PBS buffer, emission intensity dropped 8% when the pH value decreased from 7 to 5. At the same pH value, the emission intensity dropped 22% when PBS buffer concentrated from 1X to 5X.

**2.** **Photophysical measurements**

**2.1 General photophysical measurements**

The absorption spectra of the final products were measured in aqueous solution in the range 200-800 nm by an HP Agilent UV-8453 Spectrophotometer. Emission spectra from 400-1600 nm were obtained by the Fluorolog-3 TCSPC (Horiba) combined fluorescence lifetime and steady state spectrofluorometer. This is equipped with a NL-C2 Pulsed Diode Controller NanoLED, which produces picosecond and nanosecond optical pulses at a wide range of wavelengths from the ultraviolet to NIR.

**2.2 NIR Emission quantum yield**

The NIR quantum yields of **YbDD** were measured by comparison with the standard YbTPP(Tp) which was reported with a quantum yield of 3.2% in DCM with the excitation wavelength at 425 nm. ^[1-2]^ The NIR emission quantum yield of **YbDD** was calculated by the following equation:^[3]^

$\Phi_{s}=\frac{G_{s}}{G_{r}}({\frac{\eta_{s}}{\eta_{r}})}^{2}\Phi_{r}$ Equation 1

where *r* and *s* stand for the reference and sample, respectively; Φ represents the quantum yield; *G* is the slope of the plot of integrated emission intensity *vs* absorbance, and 𝜂 is the refractive index of the solvent. All spectra were corrected for the wavelength sensitivity response of the instrument.

**
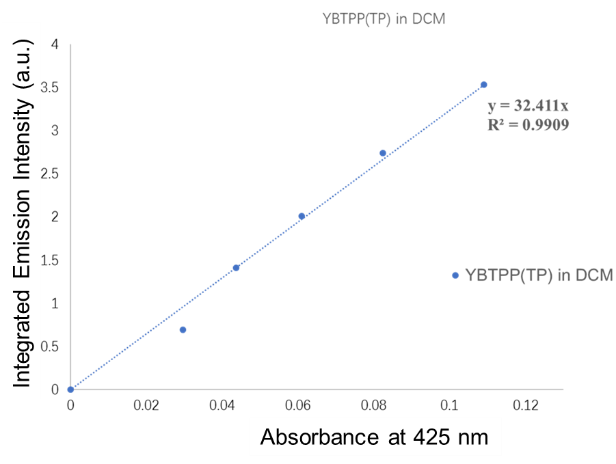
**

**

**

**
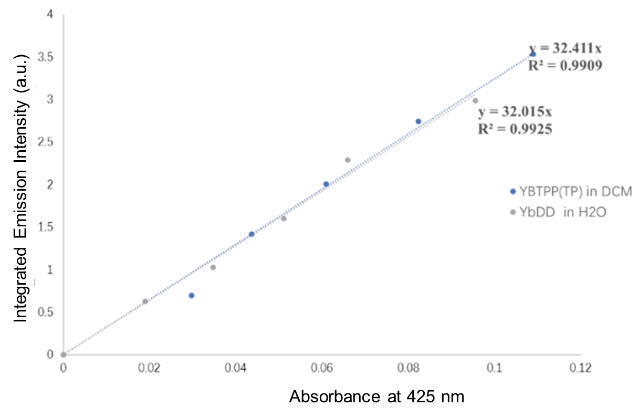

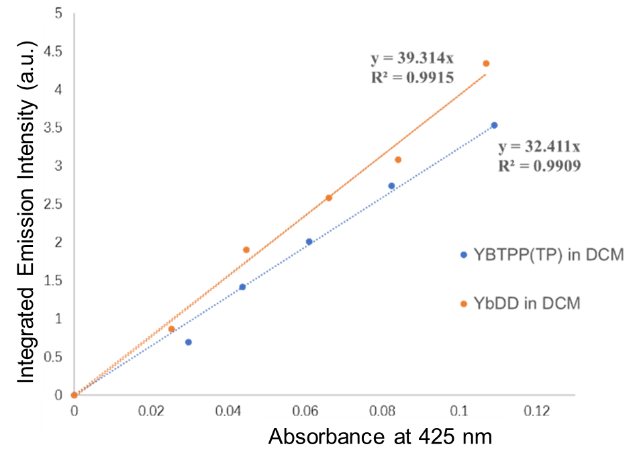


**

**

**

**Figure S11.** The NIR emission spectra of **YbDD** in DCM and water (left) and the (integrated emission *vs* absorbance) plot (right). (λ_ex_= 425nm, compared with YbTPP(Tp)).


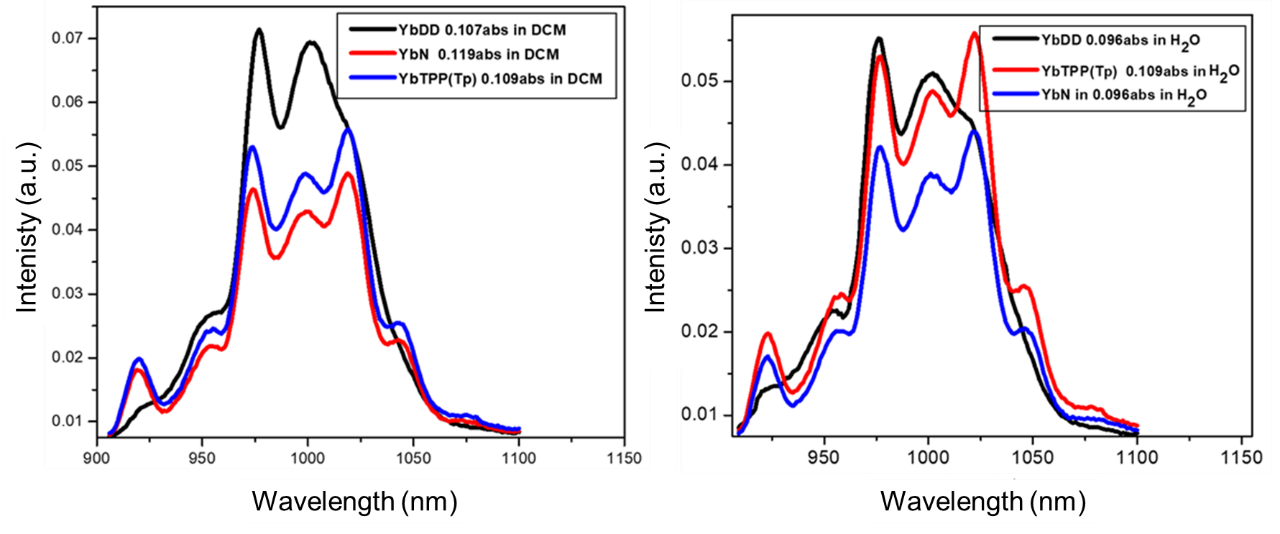


**Figure S12.** The NIR emission spectra of **YbDD** in DCM (left) and water (right) compared with **YbN** and the standard YbTPP(Tp). (λ_ex_ = 425 nm).


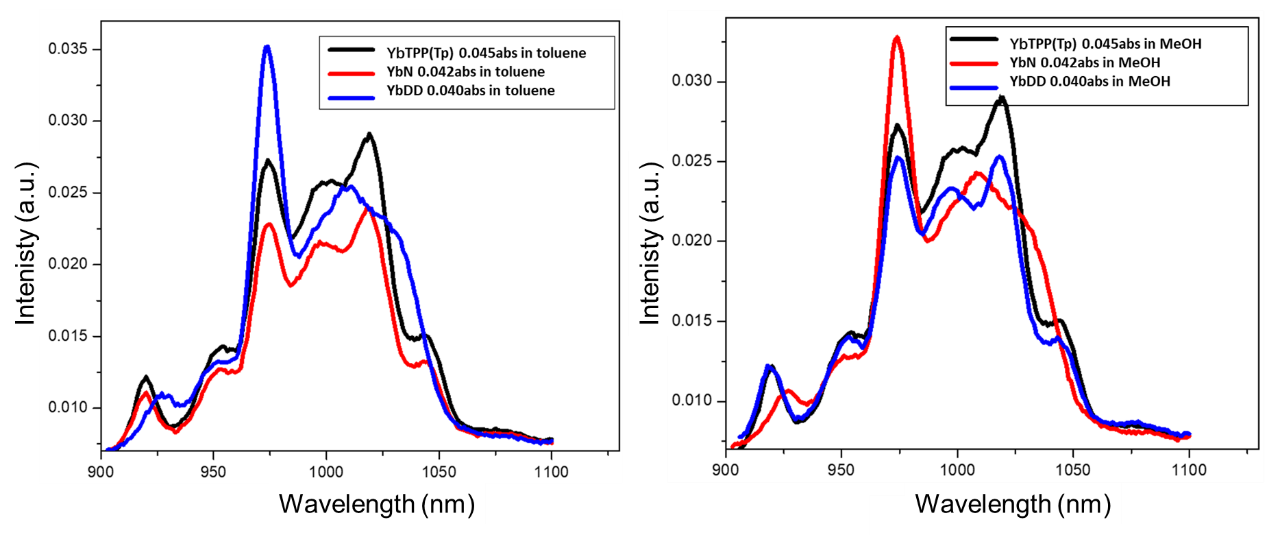


**Figure S13.** The NIR emission spectra of **YbDD** in toluene (left) and MeOH (right).

**Table S2.** The NIR emission quantum yields of **YbDD** and **YbN** in various solvents.

|  | Quantum yield (**Φ**) | | | |
| --- | --- | --- | --- | --- |
|  | DCM | H_2_O | Toluene | MeOH |
| **YbDD** | 3.9% | 2.8% | 3.5% | 2.6% |
| **YbN** | 2.8% | 2.7% | 2.8% | 2.5% |

*The NIR quantum yields of* ***YbDD*** *in DCM, H_2_O, toluene, and MeOH were measured by comparison with the standard YbTPP(Tp) in DCM with a literature reported quantum yield 3.2% with the excitation wavelength at 425 nm.*

**2.3 Photostability tests**

Photostability testing procedures follow Q1B Photostability Testing of New Drug Substances and Products admitted by U.S. Food and Drug Administration ^23^. A 1 cm quartz cell was filled with 100 µM **YbDD** solution and used as the sample “YbDD_Light”. Separately a 1 cm quartz cell was filled with the same solution, wrapped in aluminum foil to protect it completely from light, and used as the control “YbDD_Dark”. The absorption spectra for both groups were recorded as “YbDD_Light1” and “YbDD_Dark1”. The sample and control were exposed to the light source for one hour. The light source used here is HSX Xeon lamp for solar simulation (Bejing NBet Limited company). After exposure, the absorbances of the sample and the control were recorded as “YbDD_Light2” and “YbDD_Dark2”.


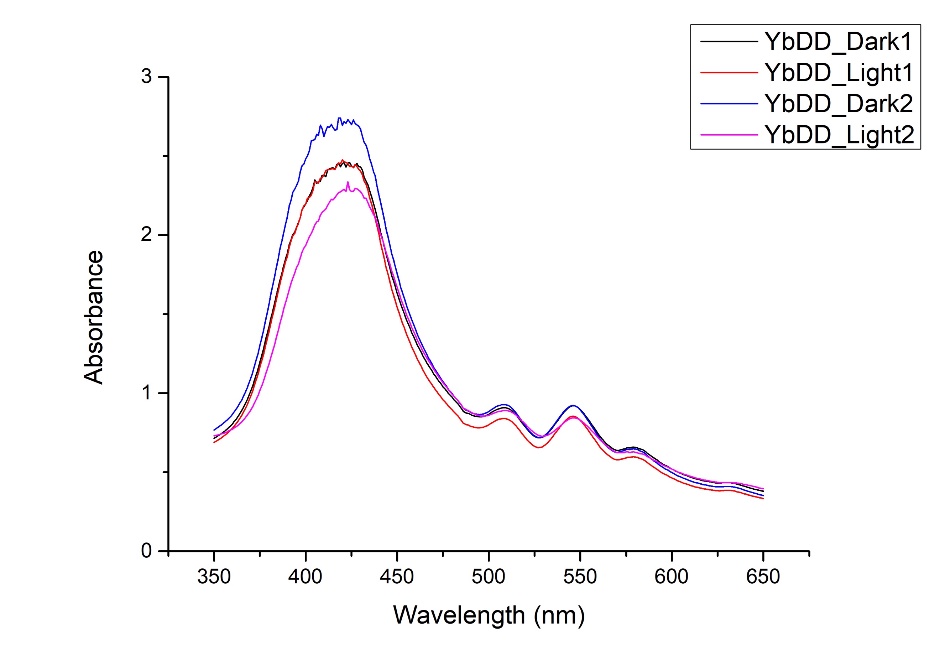


**Figure S14.** Absorption spectra of 100 µM **YbDD** aqueous solutions (with/without covering) before and after 1-hour light irradiation.

Similar changes in absorbance were observed for YbDD solutions with/without covering under light irradiation. These results prove that a common light source does not affect the photophysical properties in the time period of photodynamic applications.

**2.4 Methodology of transient absorption spectroscopy**

Helios spectrometers (Ultrafast systems, Florida, USA) were used to perform femtosecond transient absorption spectroscopy. The detailed experimental setup of the fs-TA in detail can be found in literature.^[4]^ Briefly, a white-light continuum probe pulse was generated in a 2-mm-thick sapphire plate utilizing a small fraction of the fundamental output of a Ti: sapphire femtosecond regenerative amplifier which was operating at 800 nm with 35 fs pulses and a repetition rate of 1 kHz is operated by the setup. Pump pulses at 395 nm were formed in an optical parametric amplifier (Newport Spectra-Physics). In a 2-mm-thick cuvette cell containing the sample solutions, the pump and probe pulses were overlapped temporally and spatially. The probe light transmitted from the sample was gathered and focused on a broadband UV–Vis detector to observe the change in the absorbance (∆A). The nanosecond TA spectroscopic measurements were also performed at 395 nm following laser pulse excitation. The ns-TA spectra were recorded using the pump-probe EOS setup (Ultrafast systems, Florida, USA), in which a standard probe beam is split into two: one travels through the sample, and the other one is sent directly to the reference spectrometer, which monitors the fluctuations in the probe beam intensity. The detailed experimental setup of the EOS can be found elsewhere.^[5]^

**2.5. Singlet oxygen quantum yield measurements**

**2.5.1 Singlet oxygen quantum yield in organic solvent (CHCl_3_) by emission**

Singlet oxygen was detected by its phosphorescence at 1270 nm ^[6,7]^ using the Fluorolog-3 TCSPC(HORIBA) luminescence spectrometer, and the quantum yield (Φ_Δ_) of **GdDD** in CHCl_3_ was measured through the comparison of the ^1^O_2_ emission intensity of **GdN** and a reference material (H_2_TPP, Φ_Δ_ = 0.55 in CHCl_3_) as illustrated in Eq. 2: $\Phi_{\Delta}^{S}= \Phi_{\Delta}^{R} \times\left( \frac{n_{S}}{n_{R}} \right)^{2}\frac{G_{\Delta}^{S}}{G_{\Delta}^{R}} \times\frac{A_{S}}{A_{R}}$ Eq. 2

where Φ_Δ_ denotes the singlet oxygen quantum yield; *G*_Δ_ indicates the integrated emission intensity; *A* represents the absorbance at the operation excitation wavelength; *n* reflects the solvent refractive index; and the superscripts/subscripts *R* and *S* stand for the reference and sample, respectively. In all cases, the ^1^O_2_ emission spectra were measured using excitation at 425 nm. To reduce the impact of reabsorption of the emitted light, all absorbance was controlled at 0.05.

**2.5.2 Singlet oxygen quantum yield in PBS buffer by absorption**

The 9,10-anthracenediyl-bis (methylene) dimalonic acid (ABDA) (200 μM) was mixed with a photosensitizer (10 μM) and exposed to visible light (λ = 400–700 nm) irradiation for singlet oxygen detection in PBS buffer by the absorption method. The decomposition of ABDA was monitored by the absorbance decrease at 402 nm,^[8, 9]^ which was recorded for different durations of light irradiation to obtain the decay rate of the photosensitizing process. Using Rose Bengal (RB) as a reference, the singlet oxygen quantum yield of photosensitizer (Φ_PS_) was calculated according to the following formula:

$\Phi_{PS}=\Phi_{RB}\frac{K_{PS}*A_{RB}}{K_{RB}*A_{PS}}$ Eq. 3

where *K_PS_* and *K_RB_* are the decomposition rate constants of ABDA by the photosensitizer and by RB. *A_PS_* and *A_RB_* represent the absorbed light intensity by photosensitizer and RB, which are determined by integration of the absorption bands in the wavelength range of 400–700 nm. Φ_RB_ is the singlet oxygen quantum yield of RB, which is 0.75 in water.


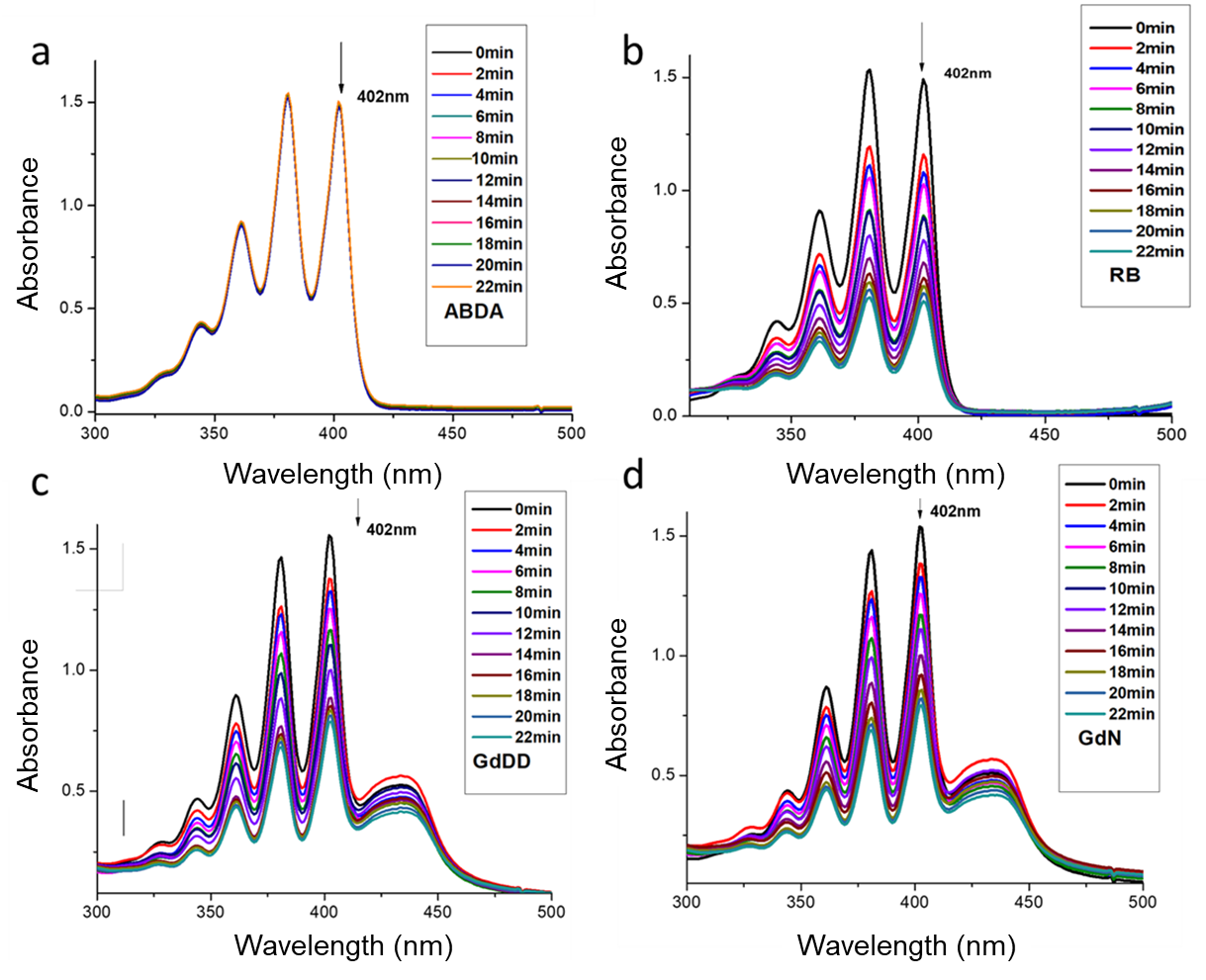


**Figure S15.** Time-dependent absorption changes of 200 μM 9,10-anthracenediyl-bis(methylene)dimalonic acid (ABDA) mixed with the probes (10 μM) a) control group b) RB, c) **GdDD** d) **GdN** in PBS buffer. The excitation power density was 6 mW cm^-2^. The instrument was equipped with a 550 nm long pass filter.


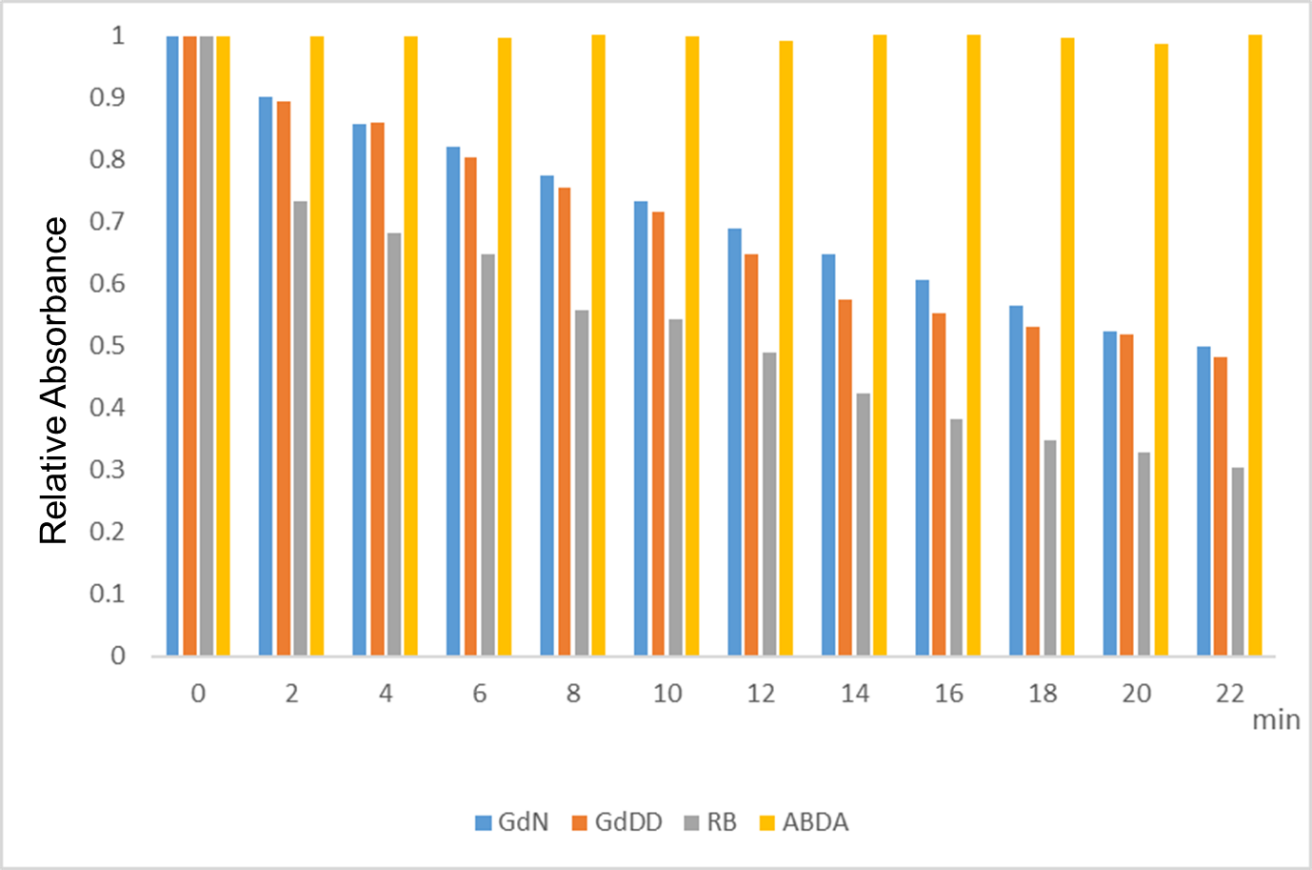


**Figure S16.** Plots of the changes in absorbance of ABDA at 402 nm (mixed with10 μM **GdDD**, GdN, and standard RB) *versus* the irradiation time (with a power density of 6 mW cm^-2^).

**2.6. Lifetime decay measurements**

The NIR emission decay of **YbDD** and of **YbN** (monitored at 978nm) in toluene and water were determined on a Fluorolog-3 Spectrofluorometer with a laser-system consisting of a Nd:YAG pump laser (1064 nm), a third-order Harmonic-Generator (355 nm, 120 mJ) and a tunable optical parameter oscillator (OPO, Spectra-Physics versaScan) with a pulse duration of 8 ns, repetition frequency of 10 Hz.



**Figure S17.** NIR emission decay of **YbDD** in water (λ_ex_ = 425 nm, Conc.: 1 μM, 298 K).





**Figure S18.** NIR emission decay of **YbN** in water (λ_ex_ = 425 nm, Conc.: 1 μM, 298 K).

**2.7 Low temperature (77 K) emission spectra measurement**

**

**Emission spectra were recorded at 77 K by employing a liquid nitrogen filled Dewar flask and Pyrex NMR tubes (4 mm diameter).

**

**

**Figure S19.** The **77** K emission spectra of **YbDD** (left) and **ErDD** (right) in MeOH (Conc.: 10 μM, λ_ex_ = 425 nm)

**

**

**Figure S20.** The 298 K absorption spectra of **LnDD** (Ln= Yb, Er, La and Gd) in aqueous solution (Conc.: 10 μM).

**



**

**Figure S21.**The visible emission (left) and NIR emission (right) spectra of **YbDD** in aqueous solution (Conc.: 1 μM, λ_ex_ = 425 nm, 298 K).

**



**

**Figure S22.** The visible emission (left) and NIR emission (right) spectra of **ErDD** in aqueous solution (Conc.: 1 μM, λ_ex_ = 425 nm, 298 K).

**

**

**Figure S23.** The emission spectrum of **GdDD** in aqueous solution (Conc.: 1 μM, λ_ex_ = 425 nm, 298 K).

**

**

**Figure S24.** The emission spectrum of **LaDD** in aqueous solution (Conc.: 1 μM, λ_ex_ = 425 nm, 298 K).

| Compound | Absorption, λ_max_, nm  (log {ε [dm^3^ mol^-1^cm^-1^]})^a^ | Emission, λ_em_, nm,^a,b^  (τ_1_,τ_2_)^c,d^ | Φ_△_^e^  in CHCl_3_  by emission | Φ_△_^f^  in PBS buffer  by absorption | Emission spectra at 77 K, λ nm^g^ (τ)^h^ |
| --- | --- | --- | --- | --- | --- |
| YbDD | 408 (5.12), 545 (4.36), 580 (4.11) | 647, 699, 923, 949, 978 (23.62 μs, 28.2 μs) 1001, 1018, 1044 | / | / | 978 (3.45 μs) ,1006, 1018 |
| ErDD | 408 (5.36), 545 (4.82), 580 (4.48) | 647, 701, 1533 (7.37 μs, /), 1602 | / | / | 1531 (/) , 1545 |
| GdDD | 412 (5.35), 545 (4.13), 580 (4.72) | 646, 703 | 0.66 | 0.46 | 744 (0.24 ms), 830, 938, 1062 |
| LaDD | 406 (5.23), 506 (3.78), 545 (4.18) | 647, 701 | / | / | / |

**Table S3.** Photophysical properties of **LnDD** (**Ln** = Yb, Er, Gd, La).

/ Not measured

[a] Absorption and emission spectra were measured in water at room temperature. [b] The visible emission standard was tetraphenylporphyrin (H_2_TPP) in anhydrous DCM (Φ_em_ = 12% at 298 K). [c] The lifetime was measured at 298 K in toluene^c^ and H_2_O ^d^. (Conc: 1 μM, λ_ex_= 425 nm) [e] The singlet oxygen quantum yield standard used in this study was tetraphenylporphyrin (H_2_TPP) in anhydrous CHCl_3_ (Φ_△_ = 55% at 298 K, λ_ex_ = 425 nm). [f] The singlet oxygen quantum yield was also evaluated in PBS buffer using RB as standard by absorption changes of ABDA at 402 nm. [g] The 77 K emission spectra were measured in MeOH (Conc.:10 μM, λ_ex_= 425 nm)). [h] The lifetime was measured at 77 K in MeOH (Conc.:10 μM, λ_ex_ = 425 nm).

**3. Photodynamic therapy studies**

**
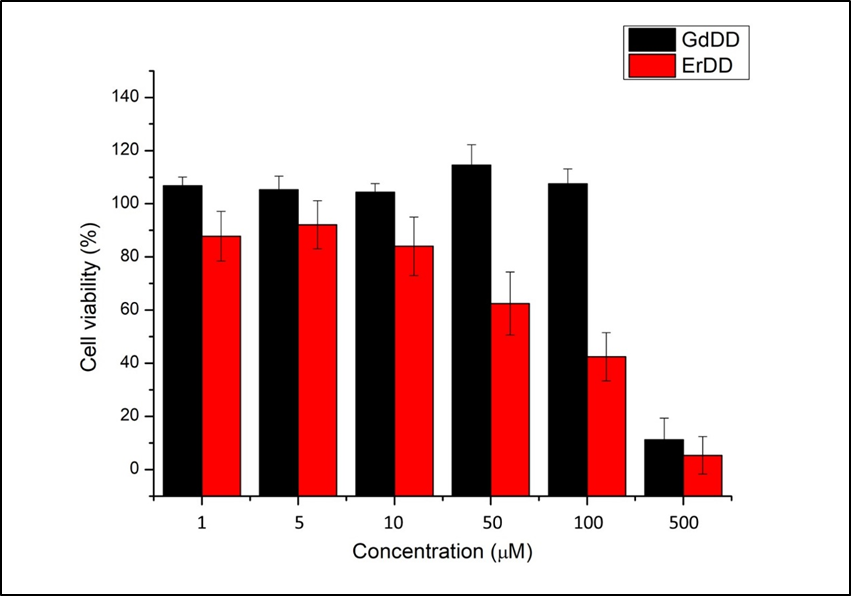
**

**Figure S25.** Dark cytotoxicity of **GdDD** and **ErDD** in HeLa cells


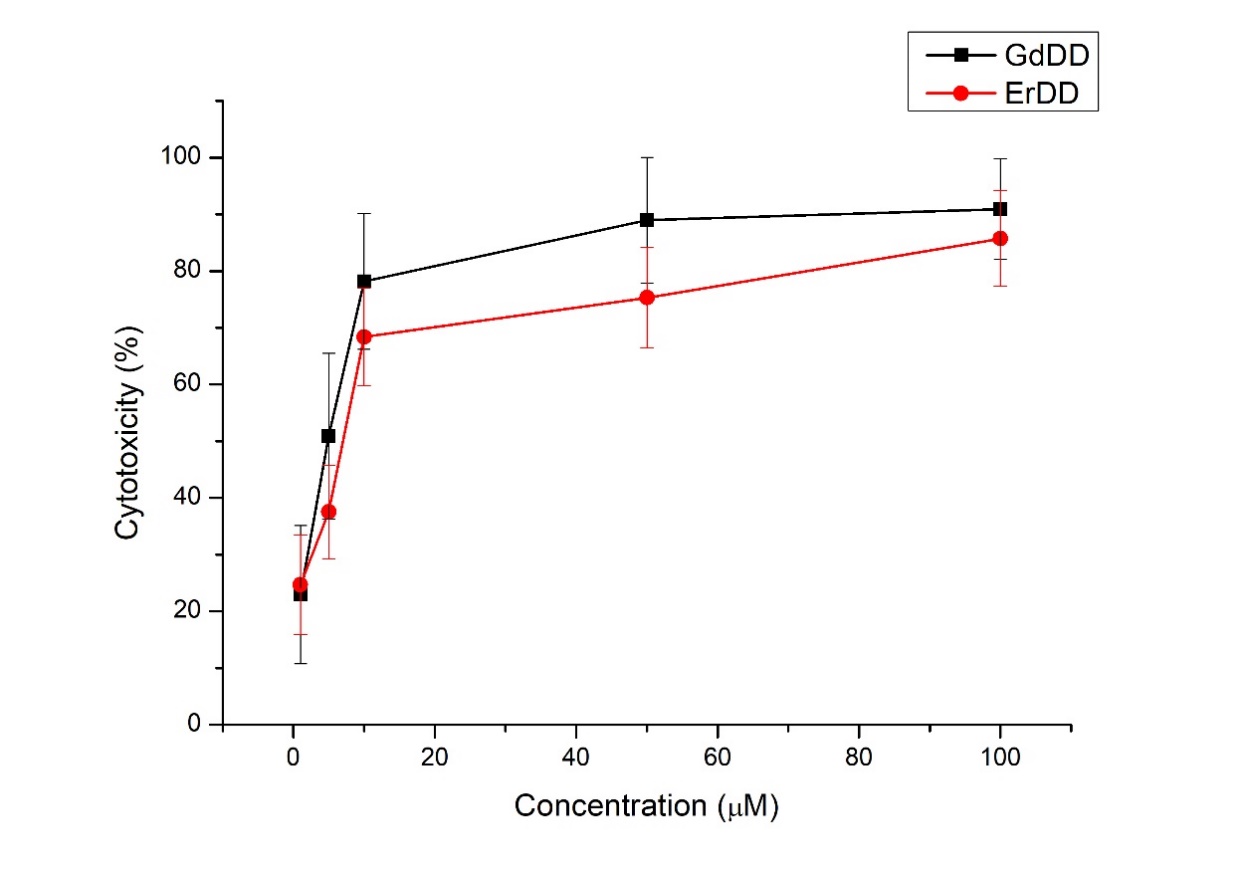


**Figure S26.** Light cytotoxicity of **GdDD** and **ErDD** in HeLa cells under light dosage of

1J/cm^2^


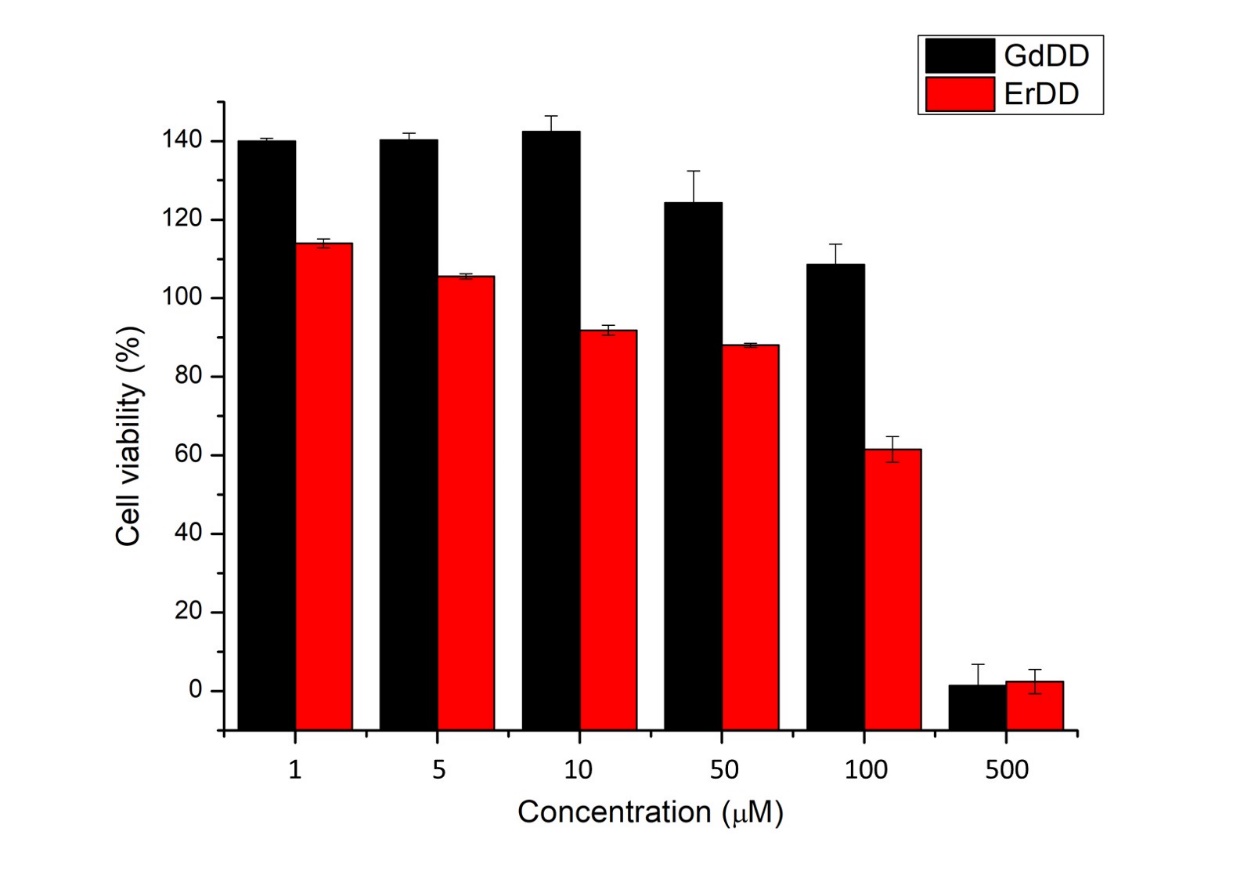
 **Figure S27.** Dark cytotoxicity of **GdDD** and **ErDD** in MRC5 cells.


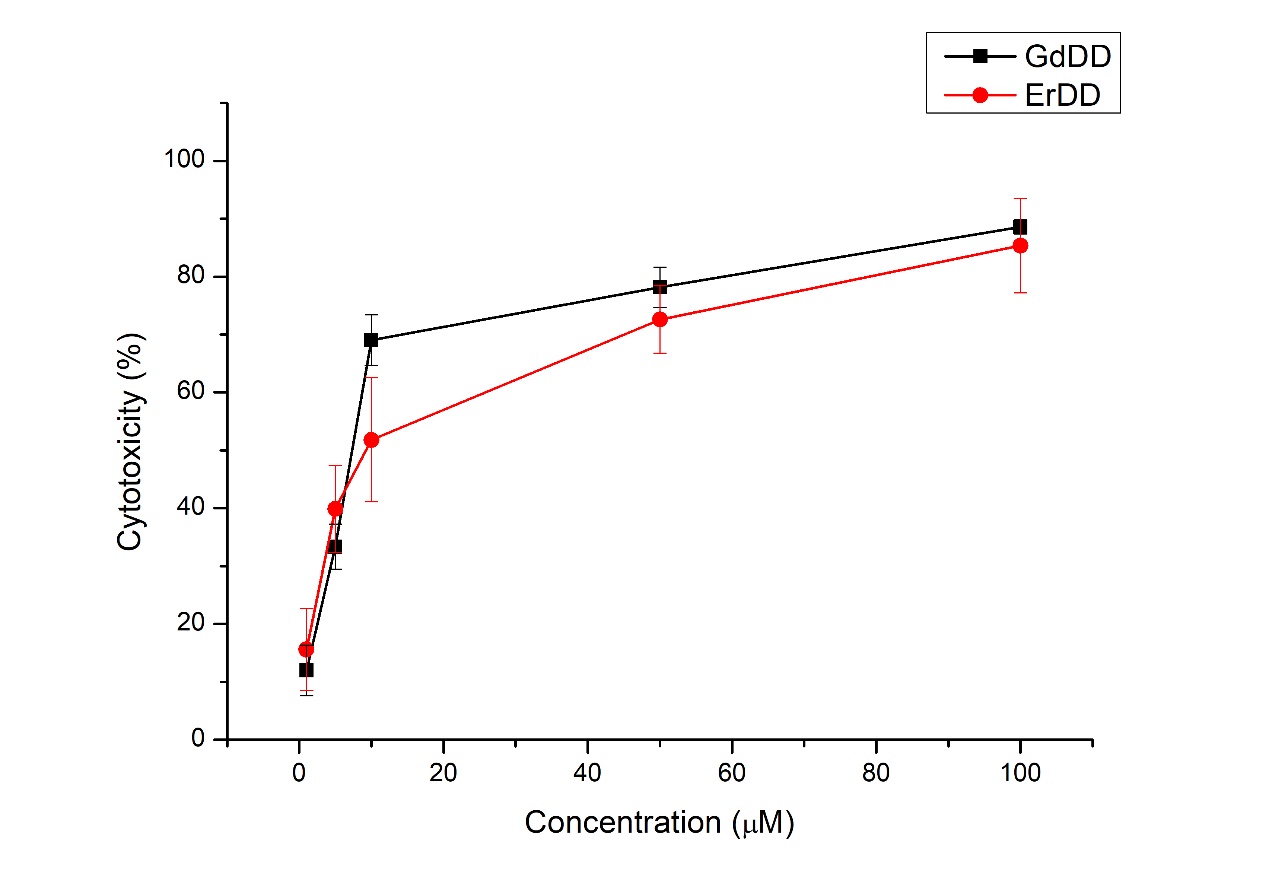


**Figure S28.** Light cytotoxicity of **GdDD** and **ErDD** in MRC5 cells under light dosage of 1J/cm^2^

**Table S4.** Photo and Dark cytotoxicity of **GdDD** and **ErDD** towards cervical cancer cell HeLa, and normal lung cell MRC5 under 1 J/cm^2^ light irradiation (λ_ex_ = 430 nm), MTT assays were carried out after incubation at 37 ^o^C for 24 h.

To evaluate the photodynamic therapeutic efficiency of **GdDD** and **ErDD**, MTT and PDT assay were carried out in HeLa (cervical cancer cells) and MRC5 (normal lung cells). Both **GdDD** and **ErDD** are safe when there is no light irradiation owing to their high dark IC_50_. Upon light irradiation, both of them showed a therapeutic effect on the cell lines. From the Table summarized above, comparisons have been made with the previously reported potential PDT agent ^22^, **GdN**. Unfortunately, exact IC_50_ values are only given for dark cytotoxicity in the reference paper, but photo-cytotoxicity can be roughly obtained from the line charts. Although two complexes have lower dark IC_50_ values than that of **GdN**, both **GdDD** and **ErDD** have similar photo IC_50_ values with **GdN**. More importantly, **GdDD** showed a large photodynamic therapeutic index which suggests its high potential as a PDT agent.

**3.2 Detection of singlet oxygen (^1^O_2_) in normaxia and hypoxia**

To detect the generation of ^1^O_2_ of GdDD in both normaxia and hypoxia conditions. PBS and GdDD (50 μM) were mixed with singlet oxygen sensor green, SOSG, (1.0 μM, dissolved in distilled water with 1% methanol) in advance. The SOSG fluorescence of each sample was tested using a fluorescence spectrometer (Horiba Fluorolog-3: λ_ex_ = 488 nm; λ_em_ = 525 nm) in both before and after the irradiation with NIR laser (808 nm; 2 W cm^-2^;1 min). For the detection of ^1^O_2_ produced in hypoxia condition, SOSG and GdDD were added into deoxygenated and sealed cuvette with deoxygenated PBS. For the detection of ^1^O_2_ produced in normaxia condition, the same experimental set up was repeated in non-sealed cuvette with PBS. Fluorescence of each sample was tested both before and after the irradiation with a NIR laser (808 nm; 2 W cm^-2^;1 min). The sample with SOSG only was set as control.


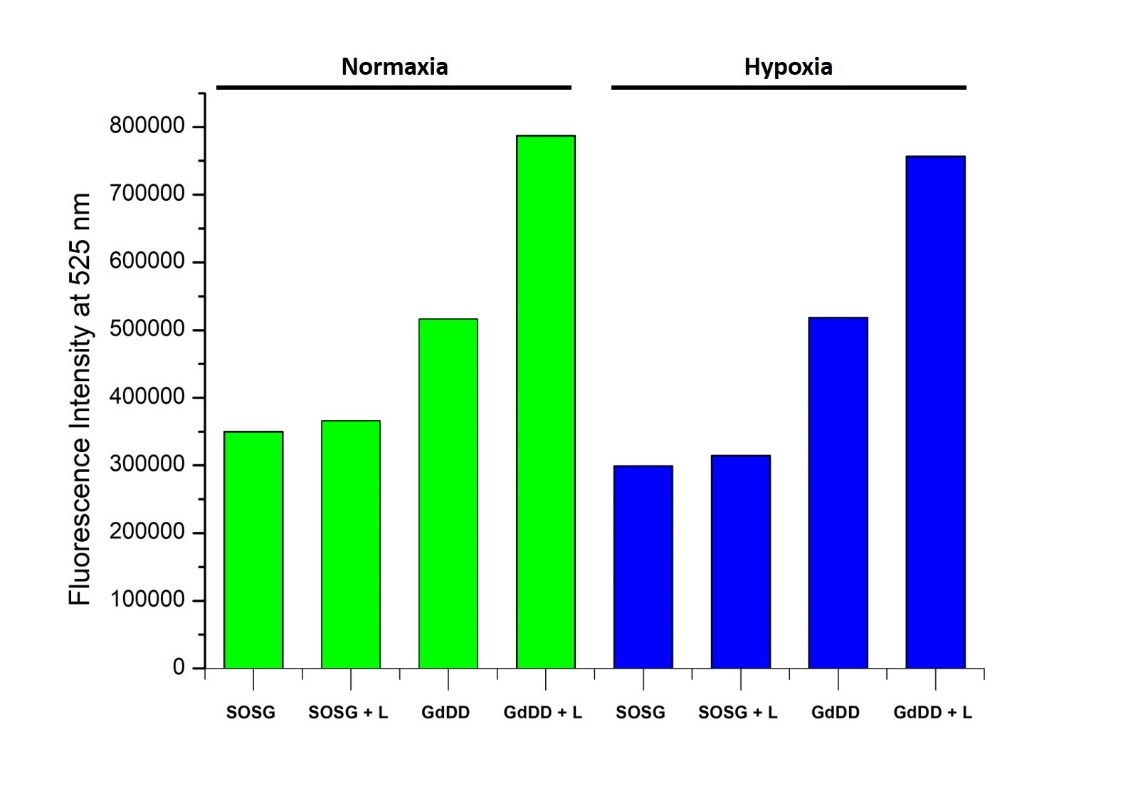


**Figure S29.** Fluorescence Intensity of emission at 525 nm of Singlet Oxygen Sensor Green of different samples in normaxia and hypoxia conditions. (L: NIR laser: 808 nm; 2 W cm^-2^; 1 min)


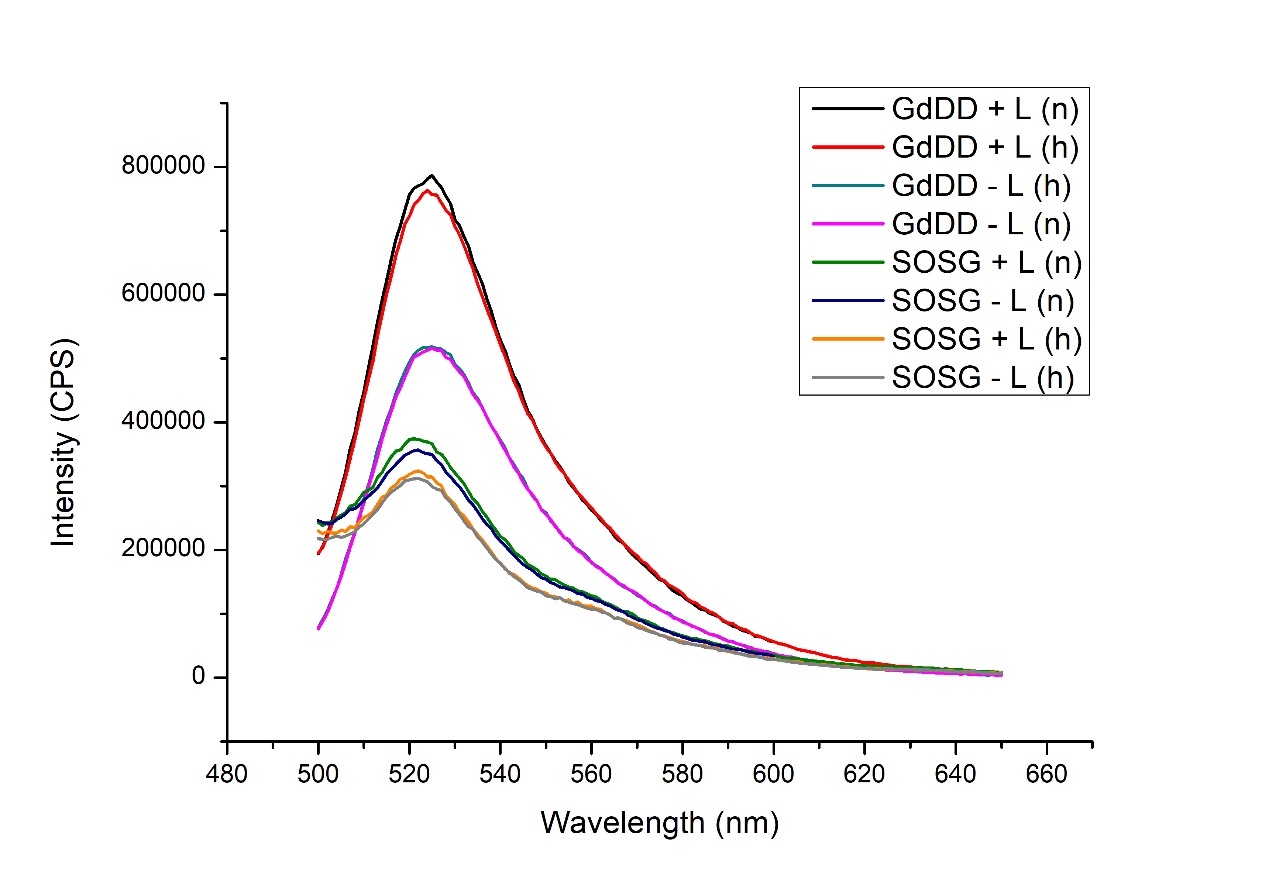


**Figure S30.** Fluorescence spectra of Singlet Oxygen Sensor Green of different samples in normaxia (n) and hypoxia conditions (h). (L: NIR laser: 808 nm; 2 W cm^-2^;1 min)

In the presence of singlet oxygen, Singlet Oxygen Sensor Green emits green fluorescence. Enhancement of emission at 525 nm of SOSG after irradiation of the NIR laser indicates the generation of singlet oxygen after NIR excitation of GdDD. The similar fluorescence intensity after NIR irradiation of GdDD demonstrates that a similar amount of singlet oxygen can be generated by GdDD in both normaxia and hypoxia conditions.

**4. Calculations**

Structures were drawn in Gabedit ^10^ and Avogadro ^11^ and saved as mol2 files. The structures were simplified by the omission of PEG sidechains. Optimizations were carried out in the vapour phase for YbDD and [YbDD]^-^ using a Lenovo Carbon pc and the results exhibited no imaginary frequencies. The geometry optimization in LUMPAC ^12^ utilized the RM1 program with standard settings in MOPAC 2016 (Molecular Orbital PACkage), a semiempirical quantum chemistry program based on Dewar and Thiel's NDDO approximation ^13^. The optimized structures are shown in Fig. S23a,b. The Module 2 in LUMPAC, Excited States Calculation, utilized the standard parameters in the Zerner's Intermediate Neglect of Differential Overlap (ZINDO/S) calculation. The calculated absorption spectrum is shown in Fig. 3a of the manuscript as a blue-dashed line.

Alternative optimizations of YbDD were performed in ORCA ^14^. For YbDD, a +3 Sparkle was substituted for Yb^3+^ (system total charge -4). The BP86 functional was employed with the Weigend def2-SVP orbital basis set ^15^ and the auxiliary basis def2/J ^16^. The optimized structure is shown in Fig. S24 and the LUMO and HOMO in Fig. S25. The calculated absorption spectrum is shown in Fig. 3a of the manuscript as a red-dotted line. The optimization of [YbDD]^-^ in ORCA used the composite approach PBeh-3c ^17^ with the basis def2-mSVP and the auxiliary basis: def2/J ^16^. The calculation uses the geometrical counterpoise correction gCP ^18^ with the atom-pairwise dispersion correction with the Becke-Johnson damping scheme (D3BJ) ^19,20^. The Firefly package^21^ was also employed to optimize the YbDD structure using the PBE0 hybrid functional (Fig. S26).

Fig. S27 displays the optimized structure in ORCA of **AlDD** using the BP86 functional with the def2-SVP basis set and auxiliary basis def2/J using Grimme's DFT-D3(BJ) robust dispersion correction method.


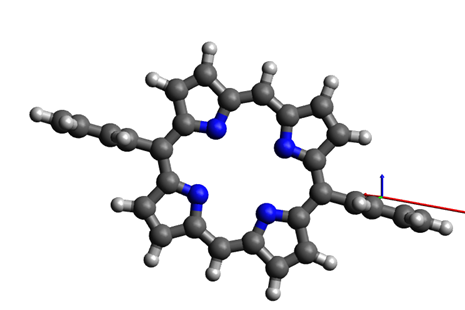


**Figure S31.** Optimized structure of the porphyrin dianion using the B3LYP functional with the Weigend def2-TZVP orbital basis set and the auxiliary basis: def2/J using a density-fitting approximation (RIJCOSX) for Coulomb integrals and numerical integration for HF exchange electron repulsion integrals. The N-C bond distance is 1.358 Å. The N-N distances are 3.028 Å and 4.195 Å. Alternatively, using the BP86 functional gives the same distances


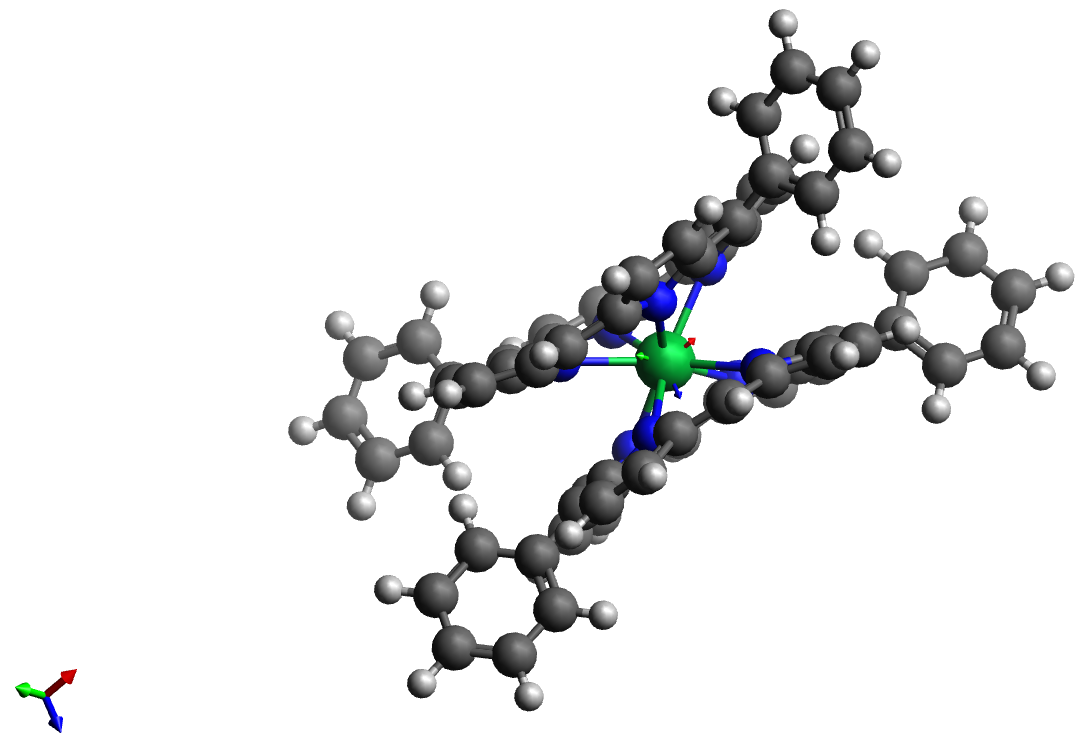


**Figure S32a.** Optimized structure of **YbDD** using the RM1 model in LUMPAC with standard settings. The LUMPAC program uses a +3 sparkle to represent Yb^3+^. The sparkle-N bond distances are in the range from 2.38-2.40 Å and the N-C distances from 1.36-1.45 Å. The in-plane N-N distances are between 2.8-4.0 Å and those from top to bottom of the double-decker are 2.8-3.0 Å.


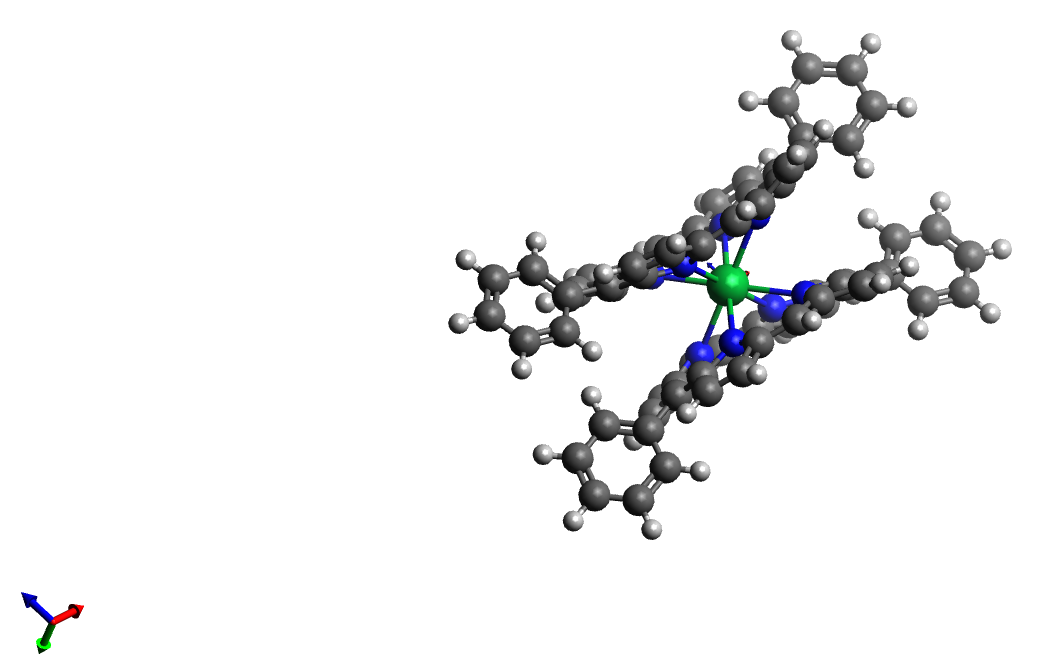


**Fig. S32b**. Optimized structure of [**YbDD]^-^** [C_64_H_40_N_8_Yb]^-^ using the RM1 model in LUMPAC with standard settings. The LUMPAC program uses a +3 sparkle to represent Yb^3+^. Yb-N distances are 2.544-2.548 Å; N-N distances 2.961-2.965 Å between layers and 2.916-2.942 Å in-layer.


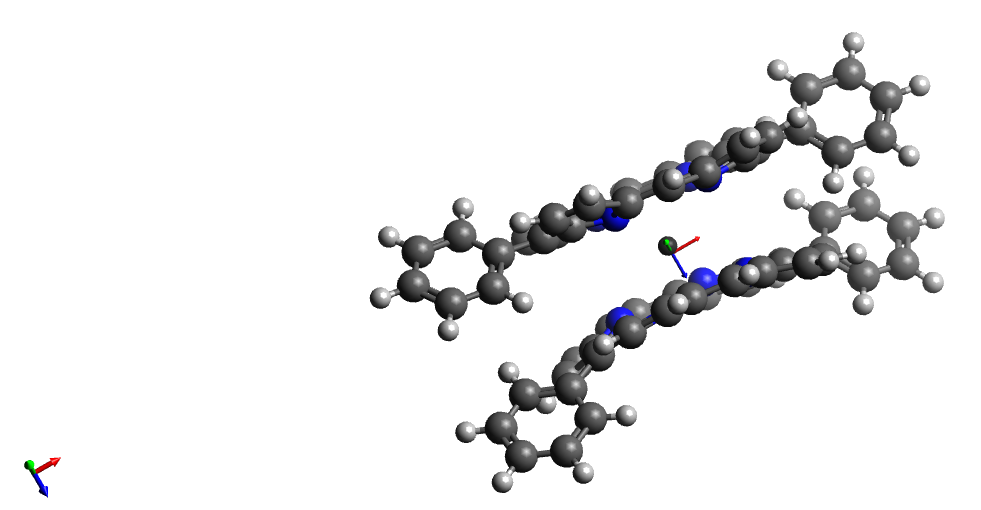


**Figure S33.** Optimized structure of **YbDD** using ORCA with the BP86 functional. The Sparkle-N distances are in the range from 2.53-2.77 Å; N-C distances from 1.369-1.376 Å and in-plane N-N distances ~3.0 Å and 4.3 Å. The top-bottom N-N distances are ~3.3 Å.


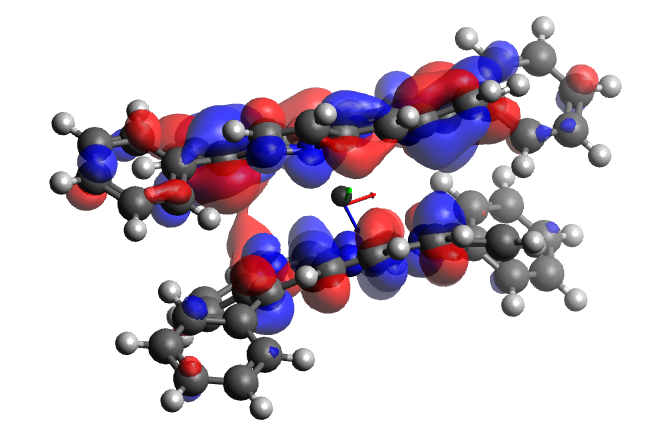

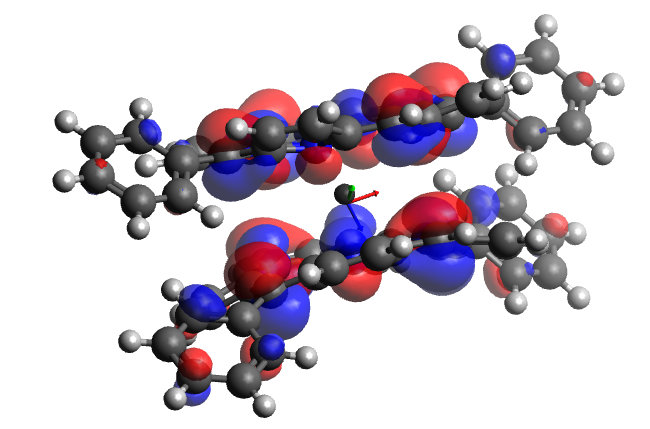


**Figure S34.** LUMO (left hand side) and HOMO (right hand side) of YbDD from the structure in Fig. S24.


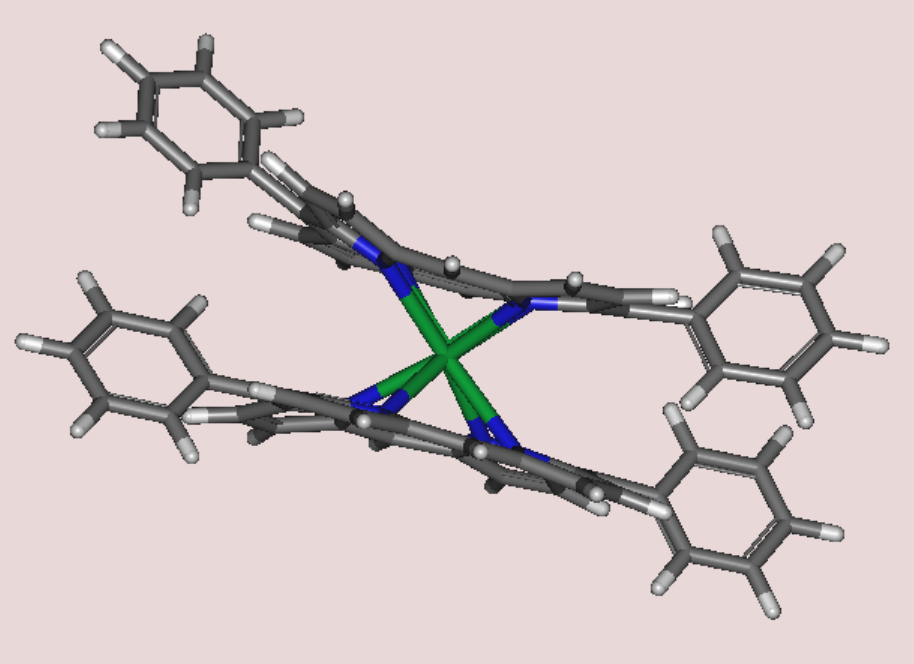


**Figure S35.** Optimized structure of **YbDD** using Firefly with the PBE0 functional. The N-N distances between the planes are between 3.23-3.30 Å.


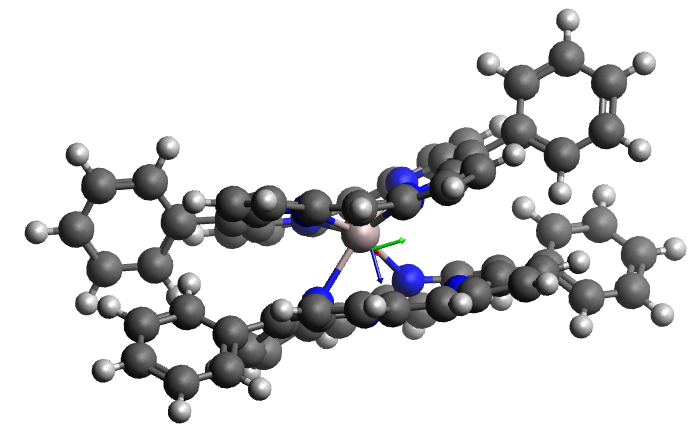


**Figure S36.** Optimized structure in ORCA of **AlDD** using the BP86 functional with the def2-SVP basis set and auxiliary basis def2/J using Grimme's DFT-D3(BJ) robust dispersion correction method. The six short Al-N distances are in the range from 2.08-2.3 Å whereas the two long distances are 3.6, 3.7 Å. The N-C distances are longer than for the **YbDD** system, being in the range 1.39-1.40 Å except for those N with longer Al-N distances (~1.36 Å in those cases). The N-N distances within the ring are between 2.8 Å and 4.2 Å, and from top to bottom of the double-decker 2.8-2.9 Å, being shorter than in **YbDD**.


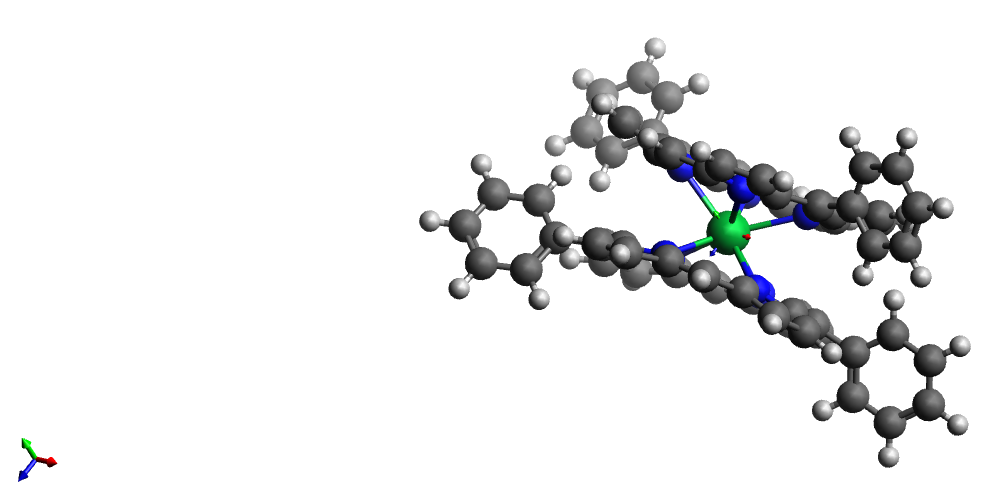


**Figure S37.** Optimized structure in ORCA of [YbDD]^-^ using the PBeh-3c functional. The Yb-N distances are 2.465±0.005 Å.

**References**

1. Foley, T. J. *et al*. *Inorg. Chem*. **42**, 5023-5032 (2003).

2. Zhang, T. *et al.* *J. Am. Chem. Soc.* **50**, 20120-21122 (2011).

3. *A Guide to Recording Fluorescence Quantum Yields*, HORIBA Jobin Yvon Inc., http://www.jobinyvon.com/usadivisions/fluorescence/applications/quantumyieldstrad. pdf

4. Bose, R. *et al*. *J. Phys. Chem. C* **119***,* 3439-3446 (2015).

5. El-Ballouli, A. a. O. *et al*. *J. Am. Chem. Soc.* **136***,* 6952-6959 (2014).

6. Zhang, J.-X. *et al*. *Bioconjugate Chem.* **23**, 1623-1638 (2012).

7. Shao, W. L. *et al*. *J. Phys. Chem. B* **116**, 14228-14234 (2012).

8. Yuan, Y. *et al.* *Chem. Sci*. **7**, 1862–1866 (2016).

9. Maria, G. B. *et al.* *J. Mater. Chem. B* **2**, 4554-4563 (2014).

10. Allouche A.-R. Gabedit, Version 2.1.0, Laboratoire de Spectrométrie Ionique et Moléculaire - UMR 5579 CNRS et Université Claude Bernard Lyon1.

11. Avogadro: an open-source molecular builder and visualization tool. Version 1.1.1. <http://avogadro.cc/>

12. Filho, M. A. M. *et al.* PLoS ONE **11**, e0154500 (2016).

13. Stewart, J. J. P. MOPAC 2016 (Molecular Orbital PACkage), MOPAC2016, Stewart Computational Chemistry, Colorado Springs, CO, USA, HTTP://OpenMOPAC.net (2016).

14. Neese, F. The ORCA program system. *Wiley Interdisciplinary Reviews: Computational Molecular Science* **2**, 73-78 (2012).

15. Weigend, F. & Ahlrichs, R. *Phys. Chem. Chem. Phys*. **7**, 3297-3305 (2005).

16. Weigend, F. *Phys. Chem. Chem. Phys.* **8**, 1057-1065 (2006).

17. Grimme, S. *et al*. *J. Chem. Phys*. **143**, 054107 (2015).

18. Kruse, H. & Grimme, S. *J. Chem. Phys.* **136**, 154101 (2012).

19. Grimme, S. *et al. J. Comput. Chem .* **32**, 1456–1465 (2011).

20. Grimme, S. *et al. J. Chem. Phys.* **132**, 154104 (2010).

21. Granovsky, A. A. Firefly package 8.2.0: <http://classic.chem.msu.su/gran/firefly/index.html>

22. Zhang, T. *et al.* In vivo selective cancer-tracking gadolinium eradicator as new-generation photodynamic therapy agent. *Proc. Natl. Acad. Sci. USA* **111,** E5492-E5497 (2014).

23. https://www.fda.gov/downloads/drugs/guidancecomplianceregulatoryinformation/guidances/ucm073373.pdf
